# A Fine-Grained Longitudinal Study of Adolescent Cannabis Use and Its Relation to Cognitive Performance in Young Adulthood

**DOI:** 10.1007/s11469-025-01604-0

**Published:** 2025-12-16

**Authors:** Clarissa Janousch, Urs Grob, Lukas Eggenberger, Lydia Johnson-Ferguson, Denis Ribeaud, Manuel Eisner, Lilly Shanahan, Boris B. Quednow

**Affiliations:** 1 Experimental Pharmacopsychology and Psychological Addiction Research, Department of Adult Psychiatry and Psychotherapy, University Hospital of Psychiatry Zurich, University of Zurich, Zurich, Switzerland; 2 Jacobs Center for Productive Youth Development, University of Zurich, Zurich, Switzerland; 3 Department of Global Public Health, Karolinska Institutet, Stockholm, Sweden; 4 Institute of Education, University of Zurich, Zurich, Switzerland; 5 Institute of Criminology, University of Cambridge, Cambridge, UK; 6 Institute of Psychology, University of Zurich, Zurich, Switzerland

**Keywords:** Addiction, Risk, Latent class growth analysis, Tetrahydrocannabinol, Marijuana, Cannabis use disorder

## Abstract

Cannabis use in adolescence has been linked to lower adult cognitive function, but the specific contributions of age of onset and usage frequency remain unclear. This study analyzed data from the *Zurich Project on Social Development from Childhood to Adulthood* (*z-proso*; *N* = 1522) to examine associations between adolescent cannabis use and cognitive performance at young adulthood. Cannabis use frequency was self-reported at six time points between ages 11 and 24. Sustained attention, spatial working memory, and visuospatial declarative memory were assessed at age 24. Regressions, repeated-measures ANOVAs with Helmert contrasts, and latent class growth analyses were conducted, all adjusted for prior cannabis use and key sociodemographic and behavioral confounders. Cannabis use at ages 13, 17, and 20 was linked to reduced attention and declarative memory at age 24, especially among almost daily/daily users. No associations were found with spatial working memory. Four usage trajectories were identified but did not differ in cognitive outcomes. Findings highlight nuanced associations, with early and frequent use linked to poorer cognitive performance.

**Supplementary Information:**

The online version contains supplementary material available at https://doi.org/10.1007/s11469-025-01604-0.

Cannabis is a psychotropic substance widely used by young people. In 2021, over 5% of 15–16-year-olds reported using it globally (United Nations Office on Drugs & Crime, 2023). In regions where recreational use has been legalized, increasing rates of cannabis use among young people have been observed. For example, the 12-month prevalence rate among US young adults reached 42.4% in 2023 (Mennis et al., 2023; Patrick et al., 2023, 2024).

During adolescence and young adulthood, the brain undergoes heightened neural development and plasticity, making it especially susceptible to the neurofunctional and neuroanatomical effects of substances (Casey & Jones, 2010; Fuhrmann et al., 2015; Gogtay et al., 2004). To explain the relationship between cannabis use and cognitive function, three key hypotheses have been proposed (Bourque & Potvin, 2021):

- The *Cognitive Vulnerability and Common Antecedent Hypothesis* suggests that preexisting cognitive profiles or common genetic and environmental factors predispose individuals to both cannabis use and cognitive difficulties.
- The *Concurrent Hypothesis* proposes that cognitive impairments occur alongside cannabis use because of the acute or post-acute effects of its primary psychoactive component, tetrahydrocannabinol (THC), making deficits more pronounced during periods of active use.
- The *Developmental Neurotoxicity Hypothesis* argues that lasting cognitive impairments result from the neurotoxic effects of cannabis exposure during sensitive developmental periods.

Adolescence and young adulthood are also characterized by enhanced sensitivity to the rewarding effects of substances (e.g., reduced inhibition, increased sense of belonging), including cannabis (Doremus-Fitzwater et al., 2010). This heightened sensitivity may increase young people’s risk of developing substance use disorders (Chen et al., 2009; Jordan & Andersen, 2017). This is particularly relevant to the *Concurrent and Developmental Neurotoxicity* hypotheses, as early and repeated exposure to cannabis during sensitive neurodevelopmental periods may not only increase the likelihood of persistent use but also contribute to cognitive impairments over time.

## Acute and Cross-Sectional Effects of Cannabis on Cognition

Experimental studies with healthy volunteers have shown that both cannabis and THC temporarily impair cognitive performance, typically with moderate effect sizes, particularly in attention, memory, and executive functions (Zhornitsky et al., 2021). Recent meta-analyses of cross-sectional studies with adult cannabis users consistently suggest that cannabis-related cognitive impairments are of small to moderate effect sizes and may be reversible with sustained abstinence (Bourque & Potvin, 2021; Scott et al., 2018). This supports the *Concurrent Hypothesis*, suggesting that although acute cognitive impairments caused by THC intoxication typically subside once the compound is fully cleared, some longer-term cognitive deficits linked to frequent cannabis use may also improve over time with sustained abstinence. This can be explained by the comparatively long detection and elimination window of THC and its metabolites, which can, in regular or heavy users, extend from several days to even weeks because of their lipophilic nature and temporary accumulation in fatty tissue (Musshoff & Madea, 2006).

Adolescents may be especially vulnerable to THC’s effects because exposure during this developmental period can induce lasting changes in brain structure (e.g., cortical thinning) and function, thereby increasing the risk of cannabis use disorder (Petrilli et al., 2022). The age of first use appears to be a critical risk factor. Several cross-sectional studies have demonstrated that early-onset users exhibit stronger cognitive deficits across various cognitive domains compared to later-onset users (for review, see Crane et al., 2013). Additionally, a cross-sectional imaging study found that initiating cannabis use before age 17 was associated with reduced overall brain volume, loss of cortical gray matter, and increased white matter volume (Wilson et al., 2000). These findings provided early support for the *Developmental Neurotoxicity Hypothesis* and highlighted the potential for lasting cognitive impairments.

## Longitudinal Findings on Cannabis Use and Cognitive Functions

In the Dunedin study, persistent cannabis use was associated with declining cognitive performance and IQ, as well as smaller hippocampal volume in midlife (Meier et al., 2012, 2022). These impairments were particularly pronounced among individuals with adolescent-onset cannabis use, although cessation largely mitigated the cognitive effects (Meier et al., 2012). In a smaller longitudinal study, combined cannabis and alcohol use between ages 16 and 19 predicted impairments in complex attention, memory, processing speed, and visuospatial functioning up to 3 years later. Moreover, an earlier age of cannabis use onset was associated with reduced processing speed and executive functioning (Jacobus et al., 2015). Another study found that the age of cannabis use onset and frequency of use during adolescence predicted reduced verbal IQ and executive functioning in early adulthood (Castellanos-Ryan et al., 2016).

However, findings have not been entirely consistent. A longitudinal U.S. twin study found that individuals with lower childhood IQ were more likely to initiate cannabis use during adolescence, and even heavy use was not associated with a decline in IQ between ages 12 and 18 (Meier et al., 2018). In the large population-based longitudinal Co-Venture study, cannabis—but not alcohol—use was associated with concurrent impairments in delayed memory recall and perceptual reasoning and sustained deficits in executive functioning (Morin et al., 2019). The IMAGEN study reported no cognitive differences among users with light, heavy, early-onset (age < 16), or late-onset (age ≥ 16) cannabis use (Wendel et al., 2021). However, the study’s cognitive battery focused on reward sensitivity, cognitive control, and emotional reactivity and did not assess delayed memory recall, which is known to be especially sensitive to chronic cannabis use (Schumann et al., 2010). Indeed, a recent longitudinal study confirmed that adolescent cannabis use was associated with declines in memory performance (Pacheco‐Colón et al., 2022). In sum, most studies—particularly larger and longitudinal ones—have found that early-onset and frequent cannabis use during adolescence is associated with longer-term changes in neurocognitive development, including disturbed mnemonic and executive functions and reduced educational attainment. These findings offer support for the *Developmental Neurotoxicity Hypothesis*.

## Cannabis Use Trajectories

The complex relationship between adolescent cannabis use and later cognitive performance may not be fully captured by linear models. Person-centered approaches more effectively account for heterogeneity in cannabis use, identifying distinct trajectories such as chronic, increasing, decreasing, infrequent, non-use, and non-regular use (e.g., Arria et al., 2015; Meier et al., 2018; Morin et al., 2019; Thompson et al., 2019).

Previous longitudinal studies have shown that frequent or chronic use is associated with lower academic performance (Silins et al., 2014), attention and memory deficits, reduced IQ (Meier et al., 2012; Morin et al., 2019), and poorer economic outcomes in adulthood (Thompson et al., 2019). These outcomes may reflect impairments in decision-making and executive functioning. Cannabis use trajectories are further influenced by sex (Crane et al., 2013; Tait et al., 2011), family background (Meier et al., 2018; Morin et al., 2019), and co-occurring substance use such as alcohol and tobacco (Jacobus et al., 2015; Meier et al., 2012; Pacheco–Colón et al., 2022).

## Current Study

Previous research on cannabis use and cognition in adolescents and young adults has several limitations. First, most existing studies do not measure cannabis use at a very young age. Second, many studies span relatively short developmental periods and are unable to assess the long-term effects of cannabis. Third, in some samples, cannabis use is not reported frequently enough during adolescence to robustly examine its associations with cognitive outcomes. Finally, some longitudinal studies have been criticized for adjusting for only a limited set of covariates (Jackson et al., 2016; Pope et al., 2003; Rogeberg, 2013).

This study addresses these limitations by examining the association between self-reported cannabis use between ages 11 and 24 and cognitive performance measured at age 24 in a community-based sample. First, we collected data on cannabis use beginning at age 11, allowing us to capture developmental trajectories prior to typical initiation. Second, we assessed cannabis use six times over a 13-year period, enabling us to track patterns across adolescence and young adulthood. Third, cannabis use was relatively common in our sample (Janousch et al., 2024; Quednow et al., 2022; Shanahan et al., 2021), allowing for robust statistical analyses of its associations with cognitive outcomes. Fourth, we employed the Cambridge Neuropsychological Test Automated Battery (CANTAB), which is grounded in neurobiologically informed cognitive constructs, offers strong interindividual translational validity, and is sensitive to both acute psychopharmacological and chronic substance use effects (Palmer et al., 2021). Finally, we assessed a comprehensive set of sociodemographic and behavioral variables, and previous cannabis use, allowing us to adjust for multiple potential confounders and thereby enhance the validity of our findings, addressing key criticisms of earlier longitudinal research (Jackson et al., 2016; Pope et al., 2003; Rogeberg, 2013). We focused on two main research questions:

(1) What is the association between self-reported cannabis use in adolescence and young adulthood and cognitive performance in young adulthood?

According to the *Developmental Neurotoxic Hypothesis*, early-onset and sustained cannabis use during adolescence is expected to result in lasting cognitive impairments due to the heightened neuroplasticity of the adolescent brain (Bourque & Potvin, 2021; Meier et al., 2012; Morin et al., 2019). Accordingly, we hypothesize that earlier initiation and higher frequency of cannabis use will be associated with poorer cognitive performance in young adulthood. Specifically, individuals who initiate cannabis use during early adolescence will exhibit lower cognitive performance at age 24 compared to those who start later or use cannabis infrequently.

In contrast, the *Concurrent Hypothesis* suggests that current cannabis use (i.e., at age 24, when cognitive performance is also assessed) is negatively associated with cognitive performance, particularly in domains such as working memory and attentional control. This is supported by prior findings indicating that cognitive impairments linked to active cannabis use may be reversible with sustained abstinence (Bourque & Potvin, 2021; Scott et al., 2018). Therefore, we hypothesize that individuals who report frequent cannabis use at age 24 will perform worse on cognitive tasks than non-users or non-frequent users, regardless of their prior use history.

(2) How are latent subgroups of cannabis use trajectories associated with cognitive performance in young adulthood?

Previous research has identified distinct cannabis use trajectories, such as chronic, increasing, decreasing, infrequent, and non-use, which are associated with differences in cognitive functioning and long-term outcomes (Arria et al., 2015; Meier et al., 2018; Morin et al., 2019; Thompson et al., 2019). Based on these findings, we hypothesize that a group of chronic early-onset cannabis users will exhibit the most pronounced cognitive impairments at age 24. Those in the increasing use group are expected to show slightly poorer cognitive performance than frequent users due to their lower exposure during earlier developmental stages. Conversely, individuals in the decreasing trajectory may show modest cognitive recovery, performing somewhat better than other user groups. Infrequent and non-users are expected to perform best cognitively, reflecting minimal disruption to neurodevelopmental processes.

## Methods

### Recruitment and Participants

Data were drawn from the *Zurich Project on Social Development from Childhood to Adulthood* (*z-proso*; Ribeaud et al., 2022). In 2004, 56 public elementary schools in Zurich were randomly selected. The targeted sample of 1675 pupils reflected the demographic composition of first-graders in Zurich’s public schools at the time due to stratification based on school size and the socioeconomic characteristics of each district. The initial assessment included 1360 children aged 7. Since 2004, eight additional assessment waves have been conducted. The sample size for the present analyses was therefore determined by the original cohort enrollment and subsequent wave-specific participation rather than by an a priori power calculation. The current analysis includes all participants who had at least one available value for self-reported cannabis use between ages 11 and 24 (assessment waves 4 to 9, *n* = 1522). Coverage for the cannabis measures was as follows: 1146 (75.3% out of 1522) at age 11, 1348 (88.6%) at age 13, 1429 (93.9%) at age 15, 1287 (84.6%) at age 17, 1178 (77.4%) at age 20, and 1158 (76.1%) at age 24. At the age of 24, 1160 participants took part in the assessment, and 1042 (89.8% out of 1160) completed the cognitive test battery. Due to the low prevalence of cannabis use at age 11, most analyses were conducted using data from wave 5 (age 13) to wave 9 (age 24; *n* = 1482). Table S1 provides an overview of participants’ demographic characteristics and compares the full sample with those from waves 5 to 9 and the cognitive subsample, revealing small, significant differences between the full sample (waves 4–9), the analytic subsample (waves 5–9), and the cognitive sample. An attrition analysis (Fig. S1) correlated wave-specific participation/missingness indicators with all cannabis-use measures (ages 11–24) and the four cognitive outcomes, using two-sided Pearson tests. Most associations were small, indicating only modest selective participation. While some differences emerged for specific covariates (e.g., demographic variables), there were no significant differences in cannabis use variables across these groups, suggesting that attrition is unlikely to account for the main findings. The *z*-proso sample is multi-ethnic and reflects Zurich’s high proportion of migrant families, with the majority of participants having at least one parent born outside of Switzerland. The study was conducted in accordance with the Declaration of Helsinki. The Cantonal Ethics Committee Zurich [BASEC #2017-02021] and the Ethics Committee of the Faculty of Arts and Social Sciences at the University of Zurich [approvals nr. Nr. 2018.2.12 and 21.12.13] approved this study. Participants provided written informed consent and received compensation for their participation in the study, appropriate to the wave and study component, ranging from CHF 30 to CHF 150. For later assessments, additional monetary compensation was provided for participation in sub-studies, including cognitive testing (CHF 40–50, depending on whether it was conducted online or in the lab). To maintain contact over the two decades of data collection, the study team employed several retention strategies, as described in detail elsewhere, including incentives to acknowledge participation, regular updates and reminders, sent through multiple channels, and flexible assessment options (Ribeaud et al., 2022; z-proso Project Team, 2024).

## Measures

### Self-Reported Cannabis Use

Frequency of cannabis use was measured with a single item. Participants were asked how often they had used cannabis in the past 12 months. Original response options ranged from 0 to 5: *never*, *once*, *2–5 times*, *6–12 times (monthly)*, *13–52 times (weekly)*, and *53–365 times (almost daily)*. Because the categories “once” and “quarterly” reflected relatively infrequent use compared to monthly, weekly, or daily use, these two categories were combined to better distinguish between occasional and regular users. As a result, the final scale consisted of five categories: *never*, *less than monthly*, *up to monthly*, *up to weekly*, and *up to daily.*

### Cognitive Functions

Cognitive performance was assessed using the Cambridge Neuropsychological Test Automated Battery (CANTAB; Sandberg, 2011), a fully computerized, nonverbal cognitive assessment tool (Strauss et al., 2006). The battery was administered either in the study laboratory or, for a minority of participants who could not attend in person, via a secure online version of the same tasks. Laboratory assessments were supervised by trained research assistants, who had been instructed in a standardized CANTAB protocol. Nonverbal tasks were chosen to ensure cultural fairness, given the high proportion of participants with migration backgrounds. Three tasks were administered: Paired Associates Learning (PAL, outcome: total errors adjusted) to measure declarative visuospatial memory, Rapid Visual Processing (RVP, outcome: A-prime) to assess sustained attention, and Spatial Working Memory (SWM, outcome: total errors) to measure visuospatial working memory. To facilitate interpretation, the error scores from the PAL and SWM tasks were inverted, with higher scores reflecting better performance. Furthermore, a total cognitive index score was calculated by averaging the *z*-standardized scores from all three tasks.

### Covariates

To address previous critiques regarding the insufficient inclusion of covariates in this area of research (Jackson et al., 2016), we included several sociodemographic and behavioral confounders, as well as previous cannabis use. Sociodemographic covariates included the following: sex assigned at birth (0 = female, 1 = male), highest completed education at age 24 (categorized as below apprenticeship, apprenticeship, vocational tertiary, and academic tertiary; recoded into dummy variables with below apprenticeship as the reference group), migration background (0 = migration background, defined as both parents born abroad; 1 = no migration background, defined as at least one parent born in Switzerland), and socioeconomic status (SES), which was measured using the International Socio-Economic Index of Occupational Status (ISEI) and *z*-standardized. This index is derived from parental occupation, ranging from 16 (e.g., unskilled worker) to 90 (e.g., judge) (Ganzeboom et al., 1992). Behavioral covariates included participants’ self-reported frequency of playing action-packed violent computer or video games (e.g., first-person shooter games, and the only gaming-related item available) in the past year, on a scale from 1 (never) to 7 (daily), given the relevance of gaming behavior when assessing cognitive functions with computerized tools (Eggenberger et al., under review). Finally, we included self-reported alcohol and tobacco consumption, assessed as past-month frequency on a 5-point Likert scale, ranging from 0 = never to 4 = (almost) daily.

## Statistical Analysis

To investigate the associations between previous and current cannabis use and young adult cognitive performance, we used the following statistical approaches: We examined direct associations between cannabis use and cognition using linear and quadratic regression models, assessing the association between cannabis use at each time point (ages 11, 13, 15, 17, 20, and 24) and cognitive performance scores at age 24. These analyses specifically test the Developmental Neurotoxic Hypothesis and the Concurrent Hypothesis by assessing whether (a) earlier or more frequent cannabis use predicts poorer cognitive performance later in life (Developmental Neurotoxic Hypothesis) and (b) current cannabis use at age 24 is associated with lower cognitive scores (Concurrent Hypothesis).

Next, to capture trends in cognitive performance across different cannabis use categories, we analyzed how frequencies of cannabis use from age 13 onward were associated with performance over time (averaged across all time points and for each time point separately). Although age 11 was included in the regression models, it was excluded from the longitudinal analyses due to the low number of users reporting cannabis use at that time. To statistically examine patterns of cannabis use over time, we employed repeated-measures ANOVAs with Helmert contrasts, which compare each factor level to the mean of all preceding levels (e.g., to daily vs. never + less than monthly + up to monthly + up to weekly). These analyses were run in R version 4.2.2 (R Core Team, 2023) with the packages *psych* (calculating psychometric properties and correlations; Revelle, 2024), *tidyverse* (transform data; Wickham et al., 2019), *car* (testing regression models; Fox & Weisberg, 2019), *rstatix* (post hoc analyses; Kassambara, 2023), and *ggplot2* (visualizing results; Wickham, 2016).

To examine developmental change in self-reported cannabis use itself, we first estimated a non-growth model and a simple latent growth curve model (LGCM, intercept-only and single-class growth). We found that these models provided weak overall fits. This suggested that a single homogeneous trajectory did not capture the observed heterogeneity in use. Therefore, beyond analyzing direct associations and category-level trends, we included a person-centered approach using latent class growth analyses (LCGAs) to examine how distinct patterns of cannabis use from adolescence to young adulthood predict cognitive outcomes. LCGAs are highly valuable because they allow us to assess heterogeneity in cannabis use patterns over time rather than treating cannabis exposure as a static variable. Given that cannabis use is known to fluctuate across development, LCGAs provide a more refined understanding of how different trajectories, rather than single time point measures, relate to cognitive function at age 24. Thus, these analyses complement the regression models by addressing whether cumulative exposure patterns differentially predict cognitive performance.

LCGAs were conducted in Mplus version 8.4 (Muthén & Muthén, 1998), using maximum likelihood with robust standard errors (MLR) and full information maximum likelihood (FIML; Cham et al., 2017) to account for missing data. Given that descriptive statistics indicated an overall increase in cannabis use up to age 20, followed by a decrease to age 24, we selected a quadratic approach, as it provided a better model fit compared to a linear approach in the LCGAs (see the Supplementary Material).

We estimated models from one to six classes and compared goodness-of-fit statistics using the Akaike Information Criterion (AIC; Akaike, 1998), the Bayesian Information Criterion (BIC; Schwarz, 1978), sample size-adjusted BIC (aBIC; Schwarz, 1978; Sclove, 1987), entropy values, the Lo–Mendell–Rubin Likelihood Ratio Test (LMR-LRT; Lo, 2001), the Vuong–Lo–Mendell–Rubin Likelihood Ratio Test (ALMR-LRT; Lo, 2001), and the Bootstrapped Likelihood Ratio Test (BLRT; McCutcheon, 1987; McLachlan & Peel, 2000; McLachlan et al., 2019).

To examine how cannabis use trajectories predict cognitive outcomes at age 24, we first applied the R3STEP procedure to assess the relationship between trajectory group membership and the sociodemographic and behavioral variables. This step allowed us to determine how external factors may influence trajectory classification and ensured a robust understanding of the characteristics associated with different cannabis use patterns. Next, we applied the Bolck–Croon–Hagenaars approach (BCH; Bolck et al., 2004) to estimate the association between cannabis use trajectories and cognitive performance while correcting for classification errors when estimating distal outcomes (Asparouhov & Muthén, 2014). Finally, to account for the sociodemographic and behavioral variables as well as for previous use in cognitive performance, we partialized out covariates from the cognitive performance scores and reran the BCH procedure using these adjusted scores. This additional step ensured that the observed cognitive differences were not solely driven by background characteristics, allowing for a clearer interpretation of the relationship between cannabis use trajectories and cognitive function.

## Results

Descriptive statistics for all study variables are reported in the Supplementary Material (Table S1). Correlations between wave-specific missingness on the cognitive outcomes (1 = missing, 0 = observed) and cannabis use at each time point were very small and mostly non-significant, indicating no systemic pattern of missingness related to cannabis use (Fig. S1). In general, cannabis use increased up to age 20 and then decreased by age 24 (Fig. S2). Figures S3–S5 illustrate the correlations, covariate-adjusted associations, and variability in cognitive performance across different levels of cannabis use.

## Variable-Centered Results

### Linear and Quadratic Regressions

**Fig. 1:**
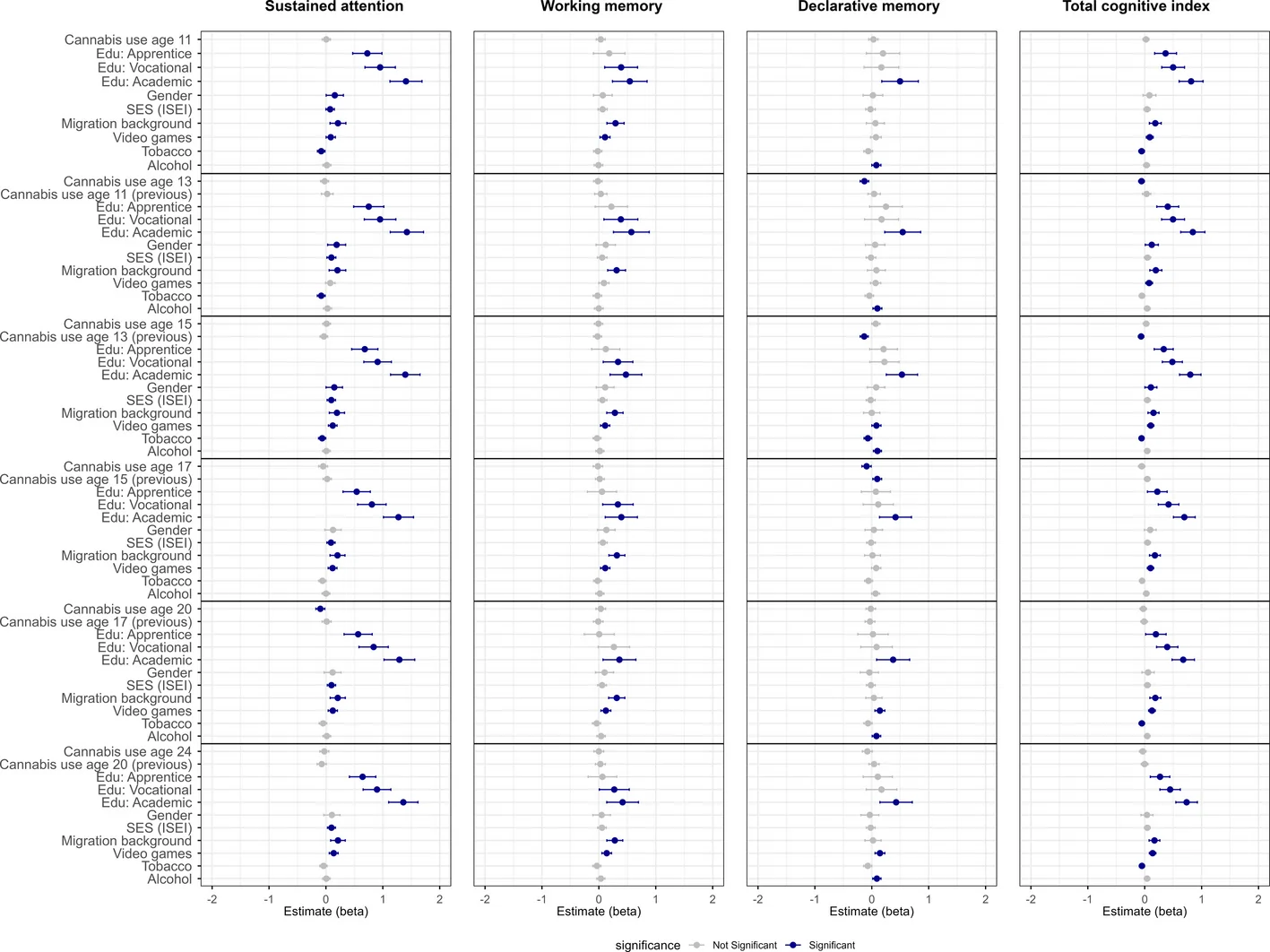
Linear regressions predicting cognitive performance from cannabis use and covariates at each time point. Note: Dots represent standardized regression coefficients (β), with 95% confidence intervals (CIs) shown as horizontal lines. Blue indicates statistically significant predictors (*p* < .05); grey indicates non-significant effects

Linear regressions adjusted for the covariates (Fig. 1, Table S2) indicated that cannabis use at some earlier developmental stages was related to lower cognitive performance at age 24, small with small effect sizes. In the fully adjusted models, cannabis use at age 13 was significantly associated with poorer declarative memory and a lower total cognitive index. Similarly, use at age 17 was significantly associated with lower declarative memory. Furthermore, cannabis use at age 20 was significantly associated with lower sustained attention, whereas use at age 24 was not significantly associated with any of the cognitive outcomes and was explained by previous use at age 20.

Quadratic regressions (Fig. S6, Table S3) did not provide substantial additional insights, as only two significant associations emerged. Significant associations were found between cannabis use at ages 13 and 15 with declarative memory. Although the developmental trajectory of cannabis use followed a quadratic pattern, rising until about age 20 and then declining, the associations between cannabis use and cognitive performance at specific time points were better described by linear models. In other words, although the development of cannabis use is best described as nonlinear, its association with cognitive outcomes at each time point is linear.

Notably, while most covariates were significantly associated with cognitive performance in the linear and quadratic regression models, almost none showed a significant association with declarative memory. This suggests that the observed relationship may be more directly attributable to cannabis use.

### Graphical Representation of Use Across Time with Cognitive Performance

**Fig. 2:**
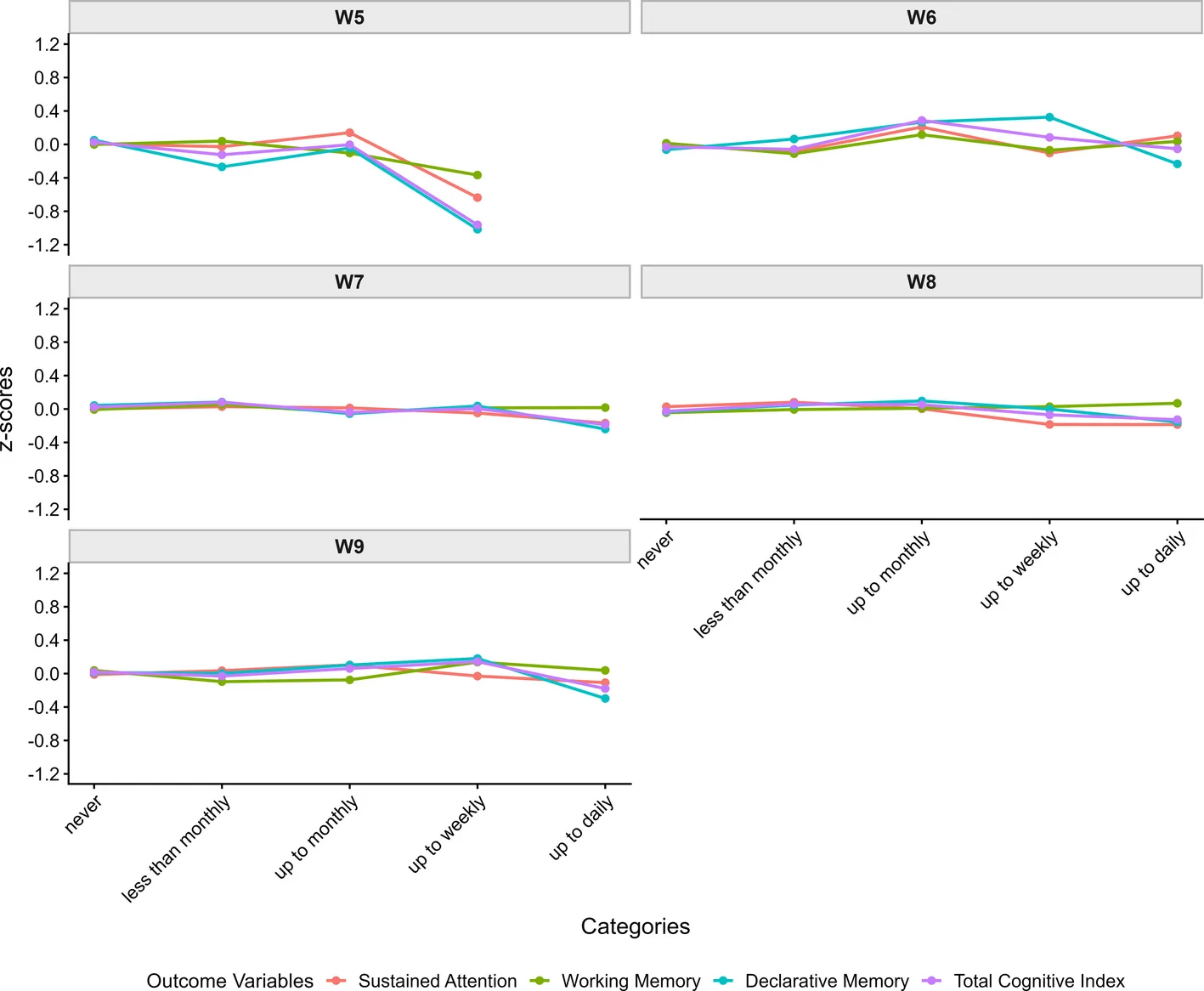
Cognitive performance (z-standardized, covariate-adjusted) by cannabis use groups for each time point. Note: The y-axis is labeled as z-scores of the cognitive outcome variables to allow for comparability across the different outcomes. The two participants reporting daily use at age 13 were excluded from this plot due to the potential for misleading associations, as these two represented highly atypical patterns

As shown in Fig. 2, cognitive performance (*y*-axis) varied according to the frequency of cannabis use (*x*-axis) across the five time points (one plot for each time point) and the three cognitive domains (colored lines). In general, cognitive performance for sustained attention, declarative memory, and total cognitive index remains relatively stable for lower cannabis use levels (*never* to *monthly use*). However, larger fluctuations and declines can be found for *up to weekly* and *up to daily* users. These trends persisted even after accounting for key sociodemographic and behavioral covariates and previous use. Thus, cannabis use frequency is linked to cognitive performance independent of background factors. No such trend was observed for working memory. The graphical representation of the relationship between use across time and the total cognitive index shows modest but consistent negative associations between higher use categories and cognitive performance.

### Repeated-Measures ANOVA Helmert Contrasts

The results of the Helmert contrasts for each CANTAB variable averaged across all time points (Table 1) confirmed the trends above. Belonging to the group of *up to daily* users indicated a significantly lower cognitive performance compared to the mean of all previous categories for declarative memory and the total cognitive index. No significant contrasts were found in working memory. Furthermore, the *up to weekly* users significantly scored lower than the previous groups in the sustained memory test. Running the Helmert contrasts for each time point separately supported this tendency (Table S4), again with small effect sizes (maximum: *η*^*2*^_partialized_ = 0.009).

**Table 1:** Helmert contrasts for each cognitive variable (averaged across all available time points) with sociodemographic covariates partialized out

	Term	Estimate	*SE*	*t*	*p*	95% CI	*η*^*2*^_partialized_
Sustained attention	Intercept	− 0.03	0.02	− 1.18	0.24	[− 0.10, 0.02]	
	Category 2 vs. 1	0.01	0.02	0.50	0.62	[− 0.03, 0.05]	0.000
	Category 3 vs. 2 + 1	0.02	0.02	0.98	0.33	[− 0.02, 0.06]	0.000
	Category 4 vs. 3 + 2 + 1	− 0.04	0.02	− 2.20	**0.03**	[− 0.07, − 0.00]	0.001
	Category 5 vs. 4 + 3 + 2 + 1	− 0.02	0.01	− 1.46	0.15	[− 0.05, − 0.01]	0.000
Working memory	Intercept	0.00	0.02	0.19	0.85	[− 0.04, 0.05]	
	Category 2 vs. 1	− 0.02	0.02	− 0.90	0.37	[− 0.05, 0.02]	0.000
	Category 3 vs. 2 + 1	0.00	0.02	0.15	0.88	[− 0.03, 0.04]	0.000
	Category 4 vs. 3 + 2 + 1	0.01	0.02	0.41	0.68	[− 0.03, 0.04]	0.000
	Category 5 vs. 4 + 3 + 2 + 1	0.01	0.01	0.62	0.54	[− 0.02, 0.04]	0.000
Declarative memory	Intercept	− 0.01	0.02	− 0.56	0.58	[− 0.06, 0.03]	
	Category 2 vs. 1	0.01	0.02	0.64	0.52	[− 0.02, 0.05]	0.000
	Category 3 vs. 2 + 1	0.02	0.02	1.01	0.31	[− 0.02, 0.06]	0.000
	Category 4 vs. 3 + 2 + 1	0.01	0.02	0.37	0.71	[− 0.03, 0.04]	0.000
	Category 5 vs. 4 + 3 + 2 + 1	− 0.06	0.01	− 3.91	** <.001**	[− 0.08, − 0.03]	0.003
Total cognitive index	Intercept	− 0.02	0.02	− 0.70	0.49	[− 0.06, 0.03]	
	Category 2 vs. 1	0.00	0.02	0.13	0.89	[− 0.03, 0.04]	0.000
	Category 3 vs. 2 + 1	0.02	0.02	1.03	0.30	[− 0.02, 0.06]	0.000
	Category 4 vs. 3 + 2 + 1	− 0.01	0.02	− 0.62	0.54	[− 0.04, 0.02]	0.000
	Category 5 vs. 4 + 3 + 2 + 1	− 0.03	0.01	− 2.30	**0.02**	[− 0.06, − 0.01]	0.001

Category 1 = never, 2 = less than monthly, 3 = up to monthly, 4 = up to weekly, 5 = up to daily; significant results are in bold

## Person-Centered Results

### Latent Class Growth Analyses

**Fig. 3:**
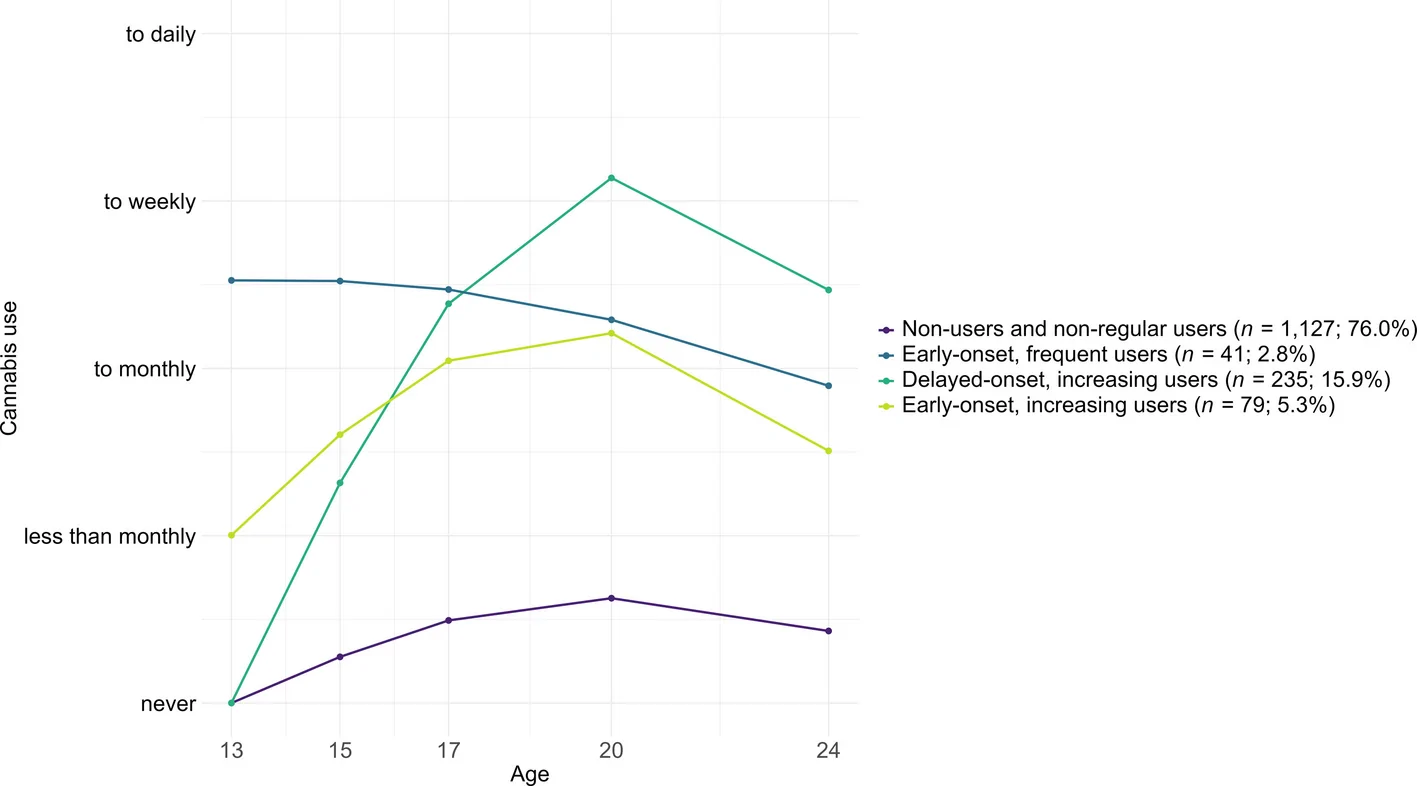
Latent Class Growth Analysis with four profiles

A no-growth and an LGCM were run for comparison. The limited fits indicate that a single-group trajectory did not adequately represent the data, thereby justifying the use of LCGA, which achieved substantially better fit values. The model fit indices supported a four-profile solution, with lower AIC, BIC, and aBIC values compared to the previous models and an entropy value of.93 (Table 2). Although AIC, BIC, and aBIC values for the five-profile solution were lower, and the entropy value for the five-profile solution was higher, we chose the four-profile solution due to theoretical implications, interpretability, good classification accuracy, and significant LMR-LR and ALMR-LR tests. The four distinct cannabis use trajectories were the groups of (1) *non-users and non-regular users* (76.0%), (2) *delayed-onset*, *increasing users* (15.9%), and the smaller groups of (3) *early-onset*, *increasing users* (5.3%), and (4) *early-onset*,* frequent users* (2.8%), as shown in Fig. 3. Although the percentage of frequent users was small, we decided, due to the large sample size, to keep these four distinctive groups.

**Table 2:** Model fit indices for mixture model analysis of cannabis use

Model		AIC	BIC	ABIC	*χ* (*p*)	*CFI*	TLI	RMSEA CI 95% [LL, UL]	SRMR		
No growth model		18,679.078	18,716.186	18,693.949	1433.695 < *.001*	.00	.01	.27 [.26,.28]	.62		
LGCM		17,221.766	17,274.777	17,243.010	906.209 < *.001*	.19	.19	.25 [.23,.26]	.18		
Model		AIC	BIC	ABIC	Entropy	LMR LR test *p*-values	ALMR LR test *p*-value	Sample proportion per class		Classification accuracy	BLRT *p*-value
LCGA	1-Class	17,944.929	17,987.338	17,961.925	1.000			1482 (100%)			
	2-Class	16,070.126	16,133.739	16,095.619	0.895	< *.001*	< *.001*	302 (20.4%); 1180 (79.6%)		.921 -.985	< *.001*
	3-Class	14,782.026	14,866.844	14,816.017	0.922	< *.001*	< *.001*	1162 (78.4%); 279 (18.8%); 41 (2.8%)		.899 -.985	< *.001*
	**4-Class**	**13,127.542**	**13,233.565**	**13,170.031**	**0.929**	***<.01***	***<.01***	**235 (15.9%); 79 (5.3%); 41 (2.8%); 1127 (76.0%)**		**.880 -.985**	** < *.001***
	5-Class	10,705.707	10,832.934	10,756.693	0.936	*.22*	*.22*	240 (16.2%); 79 (5.3%); 1122 (75.7%); 22 (1.5%); 19 (1.3%)		.859 -.983	< .001*
	6-Class	12,626.152	12,774.584	12,685.636	0.904	< *.05*	< *.05*	52 (3.5%); 1051 (70.9%); 34 (2.3%); 137 (9.2%); 43 (2.9%); 165 (11.1%)		.790 -.981	< .001*

*LGCM* Latent Growth Class Model, *LCGA* Latent Class Growth Model, *AIC* Akaike Information Criterion, *BIC* Bayesian Information Criterion, *ABIC* Adjusted Bayesian Information Criterion, *CFI* Comparative Fit Index, *TLI* Tucker-Lewis Index, *RMSEA* Root Mean Square Error of Approximation, *SRMR* Standardized Root Mean Squared Residual, *LMR LR Test* Lo-Mendell-Rubin Likelihood Ratio Test, *ALMR LR Test* Vuong-Lo-Mendell-Rubin Likelihood Ratio Test; *BLRT* Bootstrap Likelihood Ratio Test

^*^BLRT might not be trustworthy due to local maxima

**Fig. 4:**
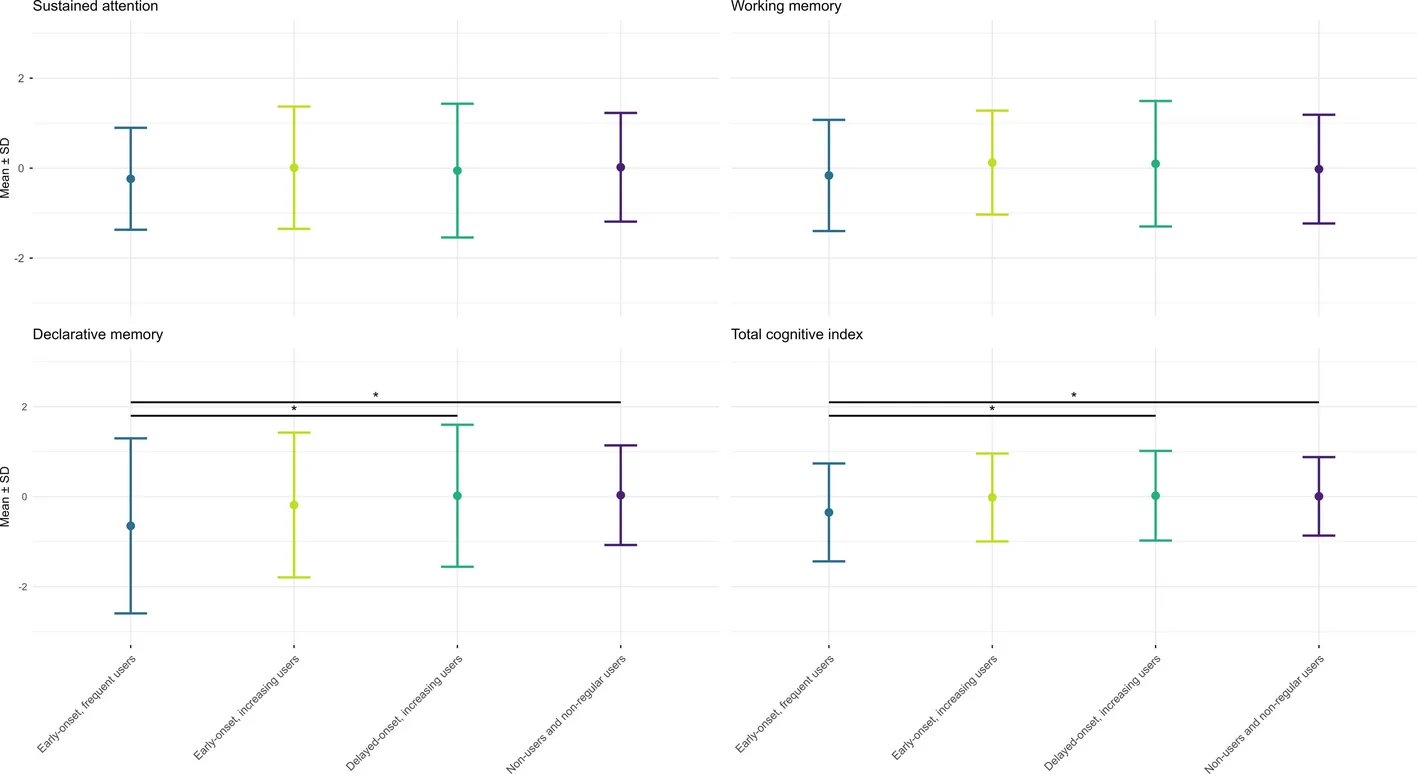
Pairwise comparisons of the cognitive scores for a 4-class solution. Note: * = *p* < .05. Scores are standardized (z-scores). Whiskers represent 95% confidence intervals for the estimated marginal means

**Fig. 5:**
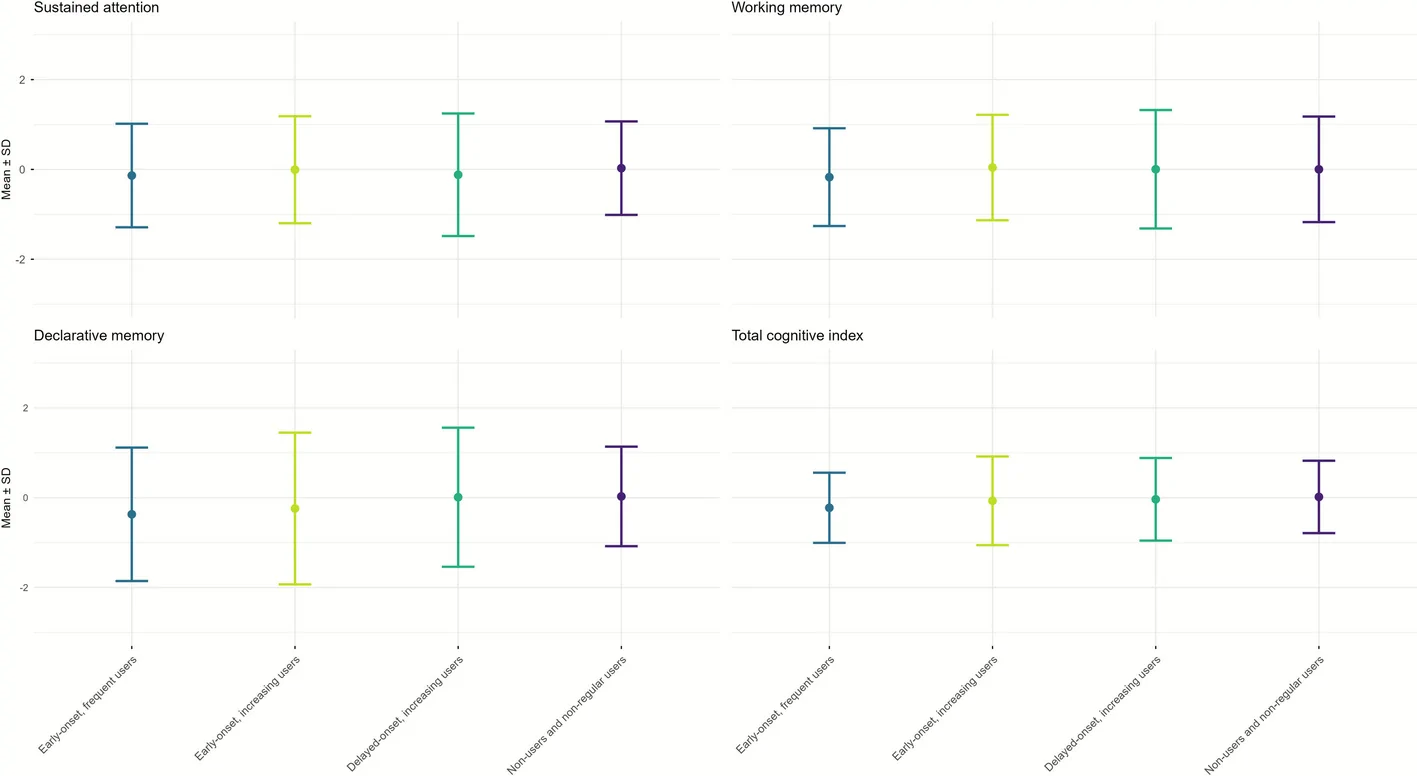
Pairwise comparisons of the cognitive scores with covariates partialized out for a 4-class solution. Note: Scores are standardized (z-scores). Whiskers represent 95% confidence intervals for the estimated marginal means

Multinomial logistic regression analyses were conducted to examine the association between group membership and the cognitive outcome variables (Fig. 4, Table S5). Participants in the early-onset, frequent user group performed significantly lower in the declarative memory task than non-users and non-regular users and delayed-onset, increasing users. This also applied to the total cognitive index. However, these associations were nonsignificant after controlling for the covariates (Fig. 5, Table S5).

**Fig. 6:**
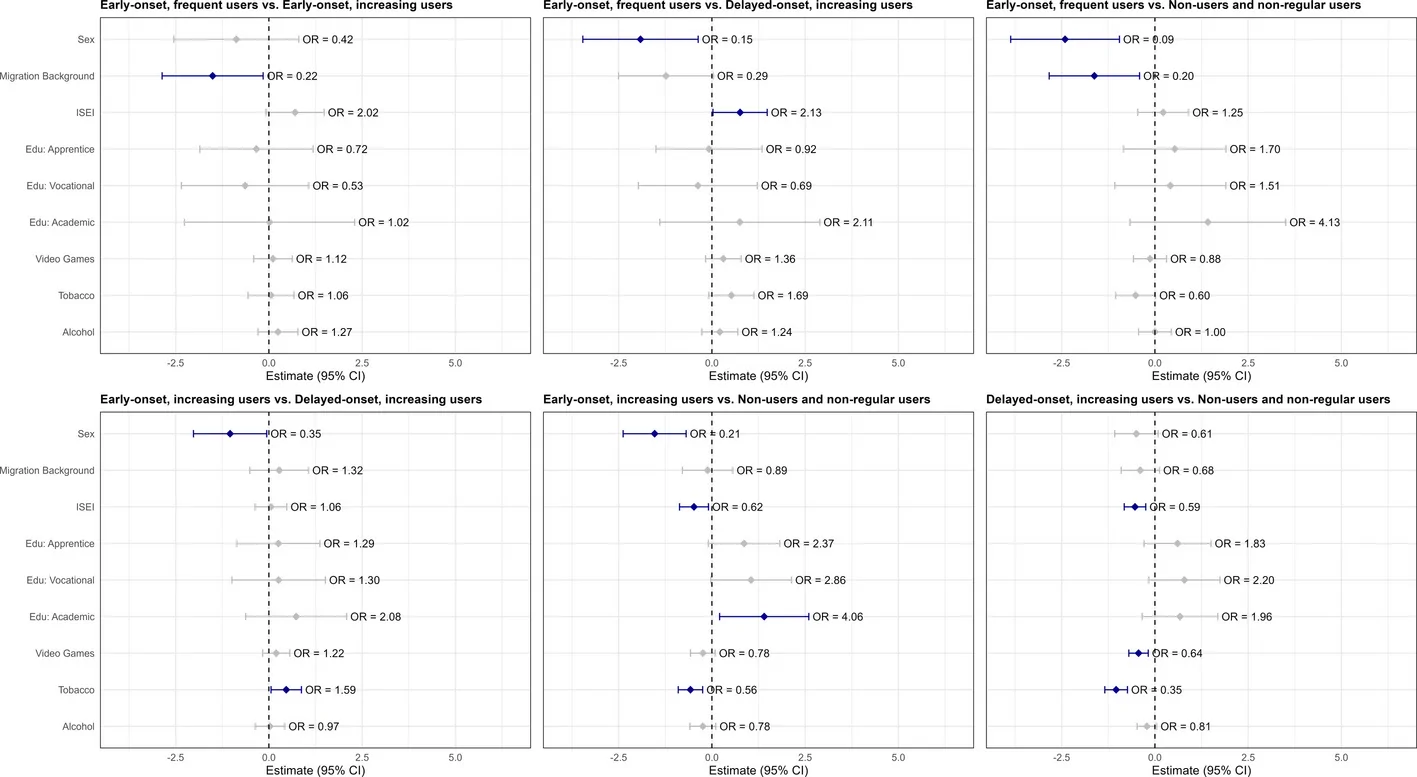
Pairwise comparisons of the sociodemographic and behavioral variables for a 4-class solution. Note: Grey lines indicate non-significance, blue indicates significance

Furthermore, we examined the relationship between the likelihood of belonging to each profile and sociodemographic and behavioral variables (Fig. 6, Table S6). Sex was a significant predictor of group membership in most pairwise comparisons, except for the early-onset, frequent users versus the early-onset, increasing users, and the delayed-onset, increasing users versus the non-users and non-regular users, indicating that males were less often part of the early-onset and more intensive use groups than females. Conversely, youth with a migration background were more likely to belong to profiles characterized by more frequent use. SES and greater educational attainment showed mixed patterns. Higher SES and education were associated with lower odds of belonging to the frequent or early-onset use profiles. However, higher education was linked to higher odds of early-onset users when compared with non-users. Education was only relevant for a few group comparisons, just like tobacco use. Behavioral factors, such as playing violent video games, differentiated some user groups from the non-user and non-regular user group, but these effects were small and not uniform across profiles.

## Discussion

This study investigated the association between cannabis use between ages 11 and 24 and cognitive functioning at age 24, using both variable-centered and person-centered approaches. After adjusting for confounding variables, the analyses revealed negative associations between frequent cannabis use at ages 13, 17, and 20 and cognitive performance at age 24—particularly in sustained attention, declarative memory, and the total cognitive index. Also, significant associations were found in the Helmert contrasts at age 24. These findings support the *Developmental Neurotoxicity* and partially the *Concurrent Hypotheses*. Working memory, by contrast, appeared less affected, showing no significant associations with cannabis use. The person-centered analyses identified four distinct user profiles: non-users and non-regular users; delayed-onset, increasing users; early-onset, increasing users; and early-onset, frequent users. However, after adjusting for sociodemographic and behavioral covariates, these user profiles were no longer significantly associated with cognitive outcomes.

Our findings support the *Developmental Neurotoxic Hypothesis*, as cannabis use at age 13 was significantly associated with declarative memory and total cognitive index scores. In particular, frequent use (*up to daily* use) was significantly associated with a lower score in cognitive functions. These associations align with previous longitudinal research, which reported that early and frequent cannabis use predicted a decline in verbal IQ and executive functioning (Jacobus et al., 2015; Meier et al., 2012). Similarly, Morin et al. (2019) highlighted frequent adolescent use predicting lasting impairments in inhibitory control. However, contrary to our findings, they also found negative associations between cannabis use and working memory. Domain-specific variability in the association between cannabis use and cognitive functions is, however, consistent with previous research, which has reported mixed findings depending on the cognitive domains (Pacheco‐Colón et al., 2022). Furthermore, meta-analyses on cross-sectional studies consistently suggest stronger effect sizes of chronic cannabis use on learning and long-term memory compared to working memory, while findings for attention remain mixed (Bourque & Potvin, 2021; Scott et al., 2018).

We only partially found support for the *Concurrent Hypothesis*. Significant associations between cannabis use and cognitive performance at age 24 were not found in the linear model; however, significant associations were found in the Helmert contrasts for declarative memory and the total cognitive index among frequent users (*up to daily use*). These results might be explained by residual effects of recent use (Zhornitsky et al., 2021), which are more likely in frequent users. However, although the data partially support the notion of concurrent effects, the single-point cognitive assessment limits our findings. We cannot confirm whether these impairments are reversible or persistent or might improve after the cessation of cannabis use, as seen in other longitudinal studies (Meier et al., 2012; Tait et al., 2011).

In general, small to moderate effect sizes were observed in this study (up to *R*^2^_adj._ = 0.25 for sustained attention in the Helmert contrasts). Notably, this aligns with previous cross-sectional studies, which have also generally reported small to moderate effects (e.g., Bourque & Potvin, 2021; Jacobus et al., 2015; Meier et al., 2018; Morin et al., 2019; Pacheco‐Colón et al., 2022; Scott et al., 2018). Factors such as metabolism, genetic predispositions, and differences in cannabis sensitivity are known to contribute to differential cognitive outcomes, potentially amplifying the problematic effects of cannabis use on cognition in specific individuals (Davis et al., 2024; Tomko et al., 2019).

The nonsignificant association between the latent groups and the cognitive outcome variables may be influenced by methodological factors. As Van Der Nest et al. (2020) have highlighted, LCGAs identify distinct trajectories within a population under the assumption of homogeneity within each class while capturing variance between classes. However, this approach does not account for within-class variability, meaning that individual differences in cannabis use patterns within the same trajectory group may still influence cognitive outcomes but remain undetected by LCGA. An alternative approach, Growth Mixture Modeling (GMM), accounts for within-class variability by incorporating random effects and could potentially yield different findings. However, GMM requires substantial statistical power, and in our study, these models failed to converge due to sample size limitations. Additionally, the measurement of self-reported cannabis use may not have been optimal. The response scale was nonlinear and possibly too coarse to capture meaningful variation in use patterns.

Importantly, the actual quantity of cannabis used was not recorded. Future studies with larger samples should aim to assess both frequency and quantity of cannabis use using interval-level scales to enhance the validity of use trajectories. Moreover, we did not collect data on the THC potency of the cannabis used, which could be a crucial factor. Given the increase in THC concentration in cannabis products in recent years, accounting for potency is important, as higher THC levels have been linked to greater risks of dependence, psychosis, and cognitive decline (Freeman et al., 2018; Potter et al., 2018). Ideally, future studies could adjust for changes in cannabis potency over time.

These findings also need to be contextualized within the unique aspects of the sample and broader research on cannabis use. This study included a community-based sample from Zurich without exclusion criteria, differing from clinical studies that typically recruit highly selective samples (Okuda et al., 2010). Additionally, Switzerland ranks among the countries with the highest national prevalence of cannabis use among adolescents and young adults, due to its de facto decriminalized status (European Monitoring Centre for Drugs and Drug Addiction (EMCDDA), 2024; Quednow et al., 2022; Shanahan et al., 2021). Due to the normalization of cannabis use in Switzerland (Sznitman, 2007; Zobel & Maier, 2018), it is prevalent across all segments of the population, making risk factors, such as low education, male sex, and migration background, only marginally relevant (Steinhoff et al., 2024).

The observed association between reduced sustained attention, as measured by the RVP task, and higher cannabis use in the most recent waves aligns with previous findings using the RVP or similar tasks in cannabis-using populations (Bechtel et al., 2022; Broyd et al., 2016; Harvey et al., 2007; Scott et al., 2018). The fact that these effects only emerged in the most recent waves may support the view that they reflect acute or post-acute effects of THC, for which the RVP is particularly sensitive (Broyd et al., 2016; Scott et al., 2018). While the association between cannabis use and visuospatial declarative memory (PAL) was significant, its relationship with spatial working memory (SWM) was not. In fact, the PAL is particularly sensitive in differentiating mild cognitive impairment from normal cognition, whereas the SWM only shows distinctions between Alzheimer’s patients, those with mild cognitive impairments, and healthy controls (Cacciamani et al., 2018; Hedayatjoo et al., 2023). This difference in sensitivity aligns with meta-analyses on cannabis and cognition, which highlight stronger impairments in long-term memory linked to hippocampal function compared to working memory (Bourque & Potvin, 2021; Ganzer et al., 2016; Scott et al., 2018). Given that PAL performance is more reliant on hippocampal integrity—and cannabis users have been found to exhibit structural abnormalities in this region (Lorenzetti et al., 2019; Meier et al., 2022)—the PAL may be particularly well-suited for detecting subtle cannabis-related cognitive deficits.

## Limitations

Several limitations of this study should be acknowledged. First, cognitive performance was assessed only once, at age 24, which limits our ability to evaluate changes in cognition over time or to establish a pre-exposure baseline. However, we included a range of covariates in our models—such as educational attainment—that are likely correlated with premorbid cognitive abilities. Furthermore, although we adjusted the models for previous cannabis use, we cannot fully disentangle acute/sub-acute effects from cumulative exposure. Consequently, findings regarding the *Concurrent Hypothesis* and the *Neurodevelopmental Hypothesis* should be considered conservative. Second, according to the cognitive reserve hypothesis (Stern, 2009), cognitive performance tends to be high in young adults (24-year-olds), potentially allowing for compensation of cannabis-related deficits. Third, we included only self-reported cannabis use, and the response scale was relatively coarse and nonlinear, which may have reduced sensitivity to detect very low or very high levels of use. However, self-reports of substance use are often characterized by high rates of underreporting, but we have previously shown that self-reported cannabis use is probably more valid compared to other illegal substances due to the normalization of cannabis use in the present population (Janousch et al., 2024; Steinhoff et al., 2023). Fourth, attrition is a significant methodological problem in longitudinal studies (Gustavson et al., 2012). Although a high number of participants remained in the study, it is possible that we lost key individuals (e.g., frequent cannabis users or those with preexisting cognitive vulnerabilities), which may have biased our findings. To address this concern, we examined missing data patterns in cognitive and cannabis use variables, as shown in the Supplementary Material (Fig. S1). We found no significant relationships between missingness and cannabis use across all time points. Finally, we did not obtain cognitive performance data from all participants.

## Conclusion

Self-reported cannabis use during adolescence and young adulthood was associated with cognitive performance in young adulthood, even after adjusting for a range of covariates. Early-onset cannabis use (age 13) as well as cannabis use at age 17 was mainly correlated with reduced declarative memory and overall cognitive functioning, whereas more recent use (at age 20) was linked to decreased sustained attention. Significant Helmert contrasts were also observed for age 24 and lower declarative memory and total cognitive index performance. Individuals who used cannabis more frequently over time generally exhibited poorer cognitive outcomes. The LCGAs identified four distinct cannabis use trajectories: *non-users and non-regular users*; *delayed-onset*, *increasing users*; *early-onset*, *increasing users*; and *early-onset*,* frequent users*. However, the association between these cannabis use trajectories and cognitive performance became nonsignificant after controlling for covariates (i.e., sex, migration background, education, SES, violent video games, alcohol, and tobacco use). This may suggest that the LCGA approach was not optimal for identifying user groups with cannabis-related cognitive impairments, as detected through regression analyses and repeated-measures ANOVAs with Helmert contrasts. In conclusion, these findings indicate that early-onset cannabis use around age 13 is linked to reduced declarative memory performance at age 24, whereas cannabis use during young adulthood is associated with diminished sustained attention at the same age.

## Supplementary Information

Below is the link to the electronic supplementary material.

ESM 1 (DOCX 1.31 MB)

## Data Availability

Anonymized data, protocols, or other material from earlier project data collections is generally available to the scientific community upon request from author DR. For data, protocols, or materials specifically from the hair or CANTAB substudies, please contact the respective principal investigators, BBQ or LS.
