# Supplementary material for: A Fine-Grained Longitudinal Study of Adolescent Cannabis Use and Its Relation to Cognitive Performance in Young Adulthood
Source: Int J Ment Health Addict. 2025 Dec 16;24(5):4119–41. doi: 10.1007/s11469-025-01604-0 (PMC13652876; doi:10.1007/s11469-025-01604-0)
Supplement: Supplementary file 1 [file 11469_2025_1604_MOESM1_ESM.docx]

**A fine-grained longitudinal study of adolescent cannabis use and its relation to
cognitive performance in young adulthood**

**SUPPLEMENT**

Janousch, C., Grob, U., Eggenberger, L., Johnson-Ferguson, L., Ribeaud, D., Eisner, M., Shanahan, L.*, Quednow, B. B.*

* contributed equally

# **Table S1.** Descriptives of all study variables.

|  | Total sample wave 4 – wave 9 (*n* = 1,522) | | | | Subsample wave 5 – wave 9 (*n* = 1,482) | | | | CANTAB sample (*n* = 1,042) | | | |  | | Total vs. subsample | | Total vs. CANTAB sample | Subsample vs. CANTAB sample | |
| --- | --- | --- | --- | --- | --- | --- | --- | --- | --- | --- | --- | --- | --- | --- | --- | --- | --- | --- | --- |
|  | *n* | *M (SD)* | Min. – max. | Skew / kurtosis | | *n* | *M (SD)* | Min. – max. | | Skew / kurtosis | *n* | *M (SD)* | Min. – max. | Skew / kurtosis | |  | Estimate (p-value) | Estimate (p-value) | Estimate (p-value) |
| **Sociodemographic covariates** |  |  |  |  | |  |  |  | |  |  |  |  |  | |  |  |  |  |
| Females, *n (%)* | 738 (48.5) | – | – | – | | 715 (48.2) | – | – | | – | 533 (51.2) | – | – | – | |  | -0.13 (.90) | 1.32 (.19) | -1.44 (.15) |
| Males | 784 (51.5) | – | – | – | | 767 (51.8) | – | – | | – | 509 (48.8) | – | – | – | |  | 0.13 (.90) | -1.32 (.19) | -1.44 (.29) |
| Household SES, *mean (SD)* | 1,436 | 45.59 (19.27) | 16 – 90 | 0.25 / 2.17 | | 1,403 | 45.74 (19.24) | 16 – 90 | | 0.25 / 2.17 | 1,042 | 47.17 (19.37) | 16 – 90 | 0.15 / 2.14 | |  | 0.21 (.84) | **2.01 (.04)** | 1.81 (.07) |
| Migration background (both parents born abroad), *n (%)* | 732 (48.1) | – | – | – | | 716 (48.3) | – | – | | – | 479 (46.0) | – | – | – | |  | 0.12 (.90) | -1.06 (0.29) | -1.16 (.25) |
| One or both parents born in Switzerland, *n (%)* | 752 (49.4) | – | – | – | | 729 (49.2) | – | – | | – | 542 (52.0) | – | – | – | |  | -0.12 (.90) | 1.30 (0.19) | 1.40 (.16) |
| Completed education, *n (%)* |  |  |  |  | |  |  |  | |  |  |  |  |  | |  |  |  |  |
| *Below apprenticeship* | 103 (6.8) | – | – | – | | 103 (6.9) | – | – | | – | 93 (8.9) | – | – | – | |  | 0.20 (.84) | **2.02 (.04)** | 1.83 (.07) |
| *Apprenticeship* | 427 (28.1) | – | – | – | | 427 (28.8) | – | – | | – | 383 (36.8) | – | – | – | |  | 0.46 (.65) | **4.65  (< .001)** | **4.21  (< .001)** |
| *Higher Vocational* | 313 (20.6) | – | – | – | | 313 (21.1) | – | – | | – | 278 (26.7) | – | – | – | |  | 0.37 (.71) | **2.61 (<.001)** | **3.25 (<.001)** |
| *Academic* | 315 (20.6) | – | – | – | | 315 (21.2) | – | – | | – | 286 (27.5) | – | – | – | |  | 0.38 (.70) | **3.96 (<.001)** | **3.60 (<.001)** |
| **Behavioral covariates** |  |  |  |  | |  |  |  | |  |  |  |  |  | |  |  |  |  |
| Playing violent video games, *n (%)* | 1,274 | 2.35 (1.76) | 1-7 | 1.13 / 3.07 | | 1,274 | 2.35 (1.76) | 1-7 | | 1.13 / 3.07 | 1,041 | 2.27 (1.69) | 1-7 | 1.24 / 3.41 | |  | 0.0 (1.0) | 1.15 (.25) | 1.15 (.25) |
| Alcohol use, *n (%)* | 1,158 | 2.04 | 0-4 | -0.50 / 2.37 | | 1,158 | 2.04 | 0-4 | | -0.50 / 2.37 | 1,040 | 2.05 (1.12) | 0-4 | -0.51 / 2.41 | |  | 0.15 (.88) | 1.30 (.19) | 1.16 (.25) |
| Tobacco use, *n (%)* | 1,156 | 1.88 | 0-4 | 0.10 / 1.26 | | 1,156 | 1.88 | 0-4 | | 0.10 / 1.26 | 1,038 | 1.89 (1.75) | 0-4 | 0.08 / 1.26 | |  | 0.42 (.67) | **4.46 (<.001)** | **4.05 (<.001)** |
| **Cannabis use** |  |  |  |  | |  |  |  | |  |  |  |  |  | |  |  |  |  |
| Cannabis use at age 11 (wave 4) | 1,146 | 1.00 (0.08) | 1 – 3 | 20.03 / 442.65 | | 1,106 | 1.00 (0.05) | 1 – 2 | | 19.12 / 366.67 | 809 | 1.00 (0.06) | 1 – 2 | 16.33 / 267.67 | |  | – | 0.00 (1.00) | 0.00 (1.00) |
| Cannabis use at age 13 (wave 5) | 1,348 | 1.18 (0.68) | 1 – 6 | 4.30 / 22.29 | | 1,348 | 1.18 (0.68) | 1 – 6 | | 4.30 / 22.29 | 962 | 1.17 (0.66) | 1 – 6 | 4.39 / 23.18 | |  | – | 0.35 (.72) | 0.35 (.72) |
| Cannabis use at age 15 (wave 6) | 1,429 | 1.90 (1.44) | 1 – 6 | 1.42 / 3.78 | | 1,429 | 1.90 (1.44) | 1 – 6 | | 1.42 / 3.78 | 1018 | 1.89 (1.43) | 1 – 6 | 1.46 / 3.96 | |  | – | 0.17 (.87) | 0.17 (.87) |
| Cannabis use at age 17 (wave 7) | 1,287 | 2.47 (1.66) | 1 – 6 | 0.70 / 2.16 | | 1,287 | 2.47 (1.66) | 1 – 6 | | 0.70 / 2.16 | 973 | 2.47 (1.64) | 1 – 6 | 0.68 / 2.18 | |  | – | 0.00 (1.00) | 0.00 (1.00) |
| Cannabis use at age 20 (wave 8) | 1,178 | 2.55 (1.67) | 1 – 6 | 0.66 / 2.14 | | 1,178 | 2.55 (1.67) | 1 – 6 | | 0.66 / 2.14 | 960 | 2.53 (1.64) | 1 – 6 | 0.67 / 2.22 | |  | – | 0.28 (.78) | 0.28 (.78) |
| Cannabis use at age 24 (wave 9) | 1,158 | 2.25 (1.64) | 1 – 6 | 1.06 / 2.85 | | 1,158 | 2.25 (1.64) | 1 – 6 | | 1.06 / 2.85 | 1040 | 2.24 (1.63) | 1 – 6 | 1.08 / 2.93 | |  | – | 0.14 (.89) | 0.14 (.89) |
| **CANTAB**  (z-standardized) |  |  |  |  | |  |  |  | |  |  |  |  |  | |  |  |  |  |
| Sustained attention | 1,041 | 0.00 (1.00) | -4.50 – 1.69 | -1.01 / 4.72 | | 1,041 | 0.00 (1.00) | -4.50 – 1.69 | | -1.01 / 4.72 | 1,041 | 0.00 (1.00) | -4.50 – 1.69 | -1.01 / 4.72 | |  | – | – | – |
| Working Memory | 1,040 | 0.00 (1.00) | -3.35 – 0.94 | -0.89 / 2.71 | | 1,040 | 0.00 (1.00) | -3.35 – 0.94 | | -0.89 / 2.71 | 1,040 | 0.00 (1.00) | -3.35 – 0.94 | -0.89 / 2.71 | |  | – | – | – |
| Declarative memory | 1,039 | 0.00 (1.00) | -5.51 – 0.85 | -2.59 / 11.45 | | 1,039 | 0.00 (1.00) | -5.51 – 0.85 | | -2.59 / 11.45 | 1,039 | 0.00 (1.00) | -5.51 – 0.85 | -2.59 / 11.45 | |  | – | – | – |
| Total cognitive index | 1,042 | 0.00 (1.00) | -4.6 – 1.57 | -1.14 / 4.62 | | 1,042 | 0.00 (1.00) | -4.6 – 1.57 | | -1.14 / 4.62 | 1,042 | 0.00 (1.00) | -4.6 – 1.57 | -1.14 / 4.62 | |  | – | – | – |

*Note*: Pairwise comparisons for metric variables were conducted using t-tests, while comparisons for proportions were performed using z-tests. Significant differences are written in bold.

# **Table S2.** Linear regressions with sociodemographic and behavioral variables as covariates for each time point.

|  | Term | Estimate | *SE* | *t* | *p* | 95% CI | Fit |
| --- | --- | --- | --- | --- | --- | --- | --- |
| Sustained attention | Intercept | -1.11 | 0.13 | -8.43 | **0.00** | [-1.37, -0.85] |  |
|  | Cannabis use at age 11 | 0.01 | 0.04 | 0.20 | 0.84 | [-0.06, 0.08] |  |
|  | Sex | 0.16 | 0.08 | 2.02 | **0.04** | [0.00, 0.31] |  |
|  | SES (ISEI) | 0.07 | 0.04 | 1.97 | 0.05 | [0.00, 0.15] |  |
|  | Migration background | 0.21 | 0.07 | 2.99 | **0.00** | [0.07, 0.35] |  |
|  | Edu: Apprenticeship | 0.73 | 0.13 | 5.57 | **0.00** | [0.47, 0.98] |  |
|  | Edu: Vocational | 0.95 | 0.14 | 7.00 | **0.00** | [0.69, 1.22] |  |
|  | Edu: Academic | 1.41 | 0.14 | 9.82 | **0.00** | [1.13, 1.69] |  |
|  | Video games | 0.08 | 0.04 | 2.04 | **0.04** | [0.00, 0.17] |  |
|  | Tobacco use | -0.08 | 0.03 | -2.44 | **0.01** | [-0.15, -0.02] |  |
|  | Alcohol use | 0.02 | 0.04 | 0.50 | 0.62 | [-0.05, 0.09] |  |
|  |  |  |  |  |  |  | R^2^ = 0.249 |
|  |  |  |  |  |  |  | R^2^_adj._ = 0.238 |
| Sustained attention | Intercept | -1.14 | 0.14 | -8.32 | **0.00** | [-1.41, -0.87] |  |
|  | Cannabis use at age 13 | -0.02 | 0.04 | -0.65 | 0.51 | [-0.09, 0.05] |  |
|  | Cannabis use at age 11 | 0.02 | 0.05 | 0.44 | 0.66 | [-0.08, 0.12] |  |
|  | Sex | 0.19 | 0.08 | 2.34 | **0.02** | [0.03, 0.35] |  |
|  | SES (ISEI) | 0.10 | 0.04 | 2.41 | **0.02** | [0.02, 0.17] |  |
|  | Migration background | 0.20 | 0.07 | 2.73 | **0.01** | [0.06, 0.35] |  |
|  | Edu: Apprenticeship | 0.75 | 0.13 | 5.58 | **0.00** | [0.49, 1.02] |  |
|  | Edu: Vocational | 0.95 | 0.14 | 6.75 | **0.00** | [0.68, 1.23] |  |
|  | Edu: Academic | 1.42 | 0.15 | 9.57 | **0.00** | [1.13, 1.71] |  |
|  | Video games | 0.08 | 0.04 | 1.80 | 0.07 | [-0.01, 0.16] |  |
|  | Tobacco use | -0.08 | 0.04 | -2.33 | **0.02** | [-0.15, -0.01] |  |
|  | Alcohol use | 0.03 | 0.04 | 0.77 | 0.44 | [-0.04, 0.10] |  |
|  |  |  |  |  |  |  | R^2^ = 0.261 |
|  |  |  |  |  |  |  | R^2^_adj._ = 0.250 |
| Sustained attention | Intercept | -1.06 | 0.12 | -8.80 | **0.00** | [-1.29, -0.82] |  |
|  | Cannabis use at age 15 | 0.01 | 0.03 | 0.36 | 0.72 | [-0.06, 0.08] |  |
|  | Cannabis use at age 13 | -0.04 | 0.03 | -1.07 | 0.29 | [-0.10, 0.03] |  |
|  | Sex | 0.15 | 0.07 | 1.99 | 0.05 | [0.00, 0.29] |  |
|  | SES (ISEI) | 0.09 | 0.04 | 2.54 | **0.01** | [0.02, 0.17] |  |
|  | Migration background | 0.19 | 0.07 | 2.84 | **0.00** | [0.06, 0.33] |  |
|  | Edu: Apprenticeship | 0.68 | 0.12 | 5.83 | **0.00** | [0.45, 0.91] |  |
|  | Edu: Vocational | 0.91 | 0.12 | 7.35 | **0.00** | [0.67, 1.15] |  |
|  | Edu: Academic | 1.40 | 0.13 | 10.57 | **0.00** | [1.14, 1.65] |  |
|  | Video games | 0.12 | 0.04 | 3.12 | **0.00** | [0.04, 0.20] |  |
|  | Tobacco use | -0.07 | 0.03 | -2.03 | **0.04** | [-0.13, 0.00] |  |
|  | Alcohol use | 0.01 | 0.03 | 0.27 | 0.79 | [-0.06, 0.08] |  |
|  |  |  |  |  |  |  | R^2^ = 0.256 |
|  |  |  |  |  |  |  | R^2^_adj._ = 0.246 |
| Sustained attention | Intercept | -0.94 | 0.12 | -7.63 | **0.00** | [-1.19, -0.70] |  |
|  | Cannabis use at age 17 | -0.05 | 0.04 | -1.24 | 0.22 | [-0.12, 0.03] |  |
|  | Cannabis use at age 15 | 0.02 | 0.04 | 0.60 | 0.55 | [-0.05, 0.09] |  |
|  | Sex | 0.12 | 0.07 | 1.69 | 0.09 | [-0.02, 0.27] |  |
|  | SES (ISEI) | 0.09 | 0.04 | 2.44 | **0.01** | [0.02, 0.16] |  |
|  | Migration background | 0.20 | 0.07 | 3.05 | **0.00** | [0.07, 0.33] |  |
|  | Edu: Apprenticeship | 0.54 | 0.12 | 4.42 | **0.00** | [0.30, 0.78] |  |
|  | Edu: Vocational | 0.81 | 0.13 | 6.33 | **0.00** | [0.56, 1.06] |  |
|  | Edu: Academic | 1.28 | 0.13 | 9.46 | **0.00** | [1.01, 1.54] |  |
|  | Video games | 0.12 | 0.04 | 2.98 | **0.00** | [0.04, 0.19] |  |
|  | Tobacco use | -0.06 | 0.03 | -1.85 | 0.07 | [-0.13, 0.00] |  |
|  | Alcohol use | 0.00 | 0.03 | 0.06 | 0.95 | [-0.07, 0.07] |  |
|  |  |  |  |  |  |  | R^2^ = 0.237 |
|  |  |  |  |  |  |  | R^2^_adj._ = 0.228 |
| Sustained attention | Intercept | -0.96 | 0.13 | -7.56 | **0.00** | [-1.21, -0.71] |  |
|  | Cannabis use at age 20 | -0.10 | 0.04 | -2.46 | **0.01** | [-0.18, -0.02] |  |
|  | Cannabis use at age 17 | 0.01 | 0.04 | 0.32 | 0.75 | [-0.07, 0.09] |  |
|  | Sex | 0.12 | 0.08 | 1.55 | 0.12 | [-0.03, 0.26] |  |
|  | SES (ISEI) | 0.10 | 0.04 | 2.65 | **0.01** | [0.03, 0.17] |  |
|  | Migration background | 0.21 | 0.07 | 3.08 | **0.00** | [0.08, 0.34] |  |
|  | Edu: Apprenticeship | 0.57 | 0.13 | 4.49 | **0.00** | [0.30, 0.81] |  |
|  | Edu: Vocational | 0.84 | 0.13 | 6.39 | **0.00** | [0.56, 1.10] |  |
|  | Edu: Academic | 1.29 | 0.14 | 9.39 | **0.00** | [1.02, 1.56] |  |
|  | Video games | 0.12 | 0.04 | 2.94 | **0.00** | [0.04, 0.20] |  |
|  | Tobacco use | -0.05 | 0.03 | -1.49 | 0.14 | [-0.12, 0.02] |  |
|  | Alcohol use | 0.02 | 0.03 | 0.44 | 0.66 | [-0.05, 0.08] |  |
|  |  |  |  |  |  |  | R^2^ = 0.252 |
|  |  |  |  |  |  |  | R^2^_adj._ = 0.242 |
| Sustained attention | Intercept | -1.00 | 0.12 | -8.41 | **0.00** | [-1.21, -0.71] |  |
|  | Cannabis use at age 24 | -0.03 | 0.04 | -0.69 | 0.49 | [-0.18, -0.02] |  |
|  | Cannabis use at age 20 | -0.07 | 0.04 | -1.79 | 0.07 | [-0.07, 0.09] |  |
|  | Sex | 0.10 | 0.07 | 1.45 | 0.15 | [-0.03, 0.26] |  |
|  | SES (ISEI) | 0.10 | 0.04 | 2.74 | **0.01** | [0.03, 0.17] |  |
|  | Migration background | 0.21 | 0.07 | 3.23 | **0.00** | [0.08, 0.34] |  |
|  | Edu: Apprenticeship | 0.64 | 0.12 | 5.45 | **0.00** | [0.41, 0.88] |  |
|  | Edu: Vocational | 0.90 | 0.12 | 7.24 | **0.00** | [0.65, 1.14] |  |
|  | Edu: Academic | 1.36 | 0.13 | 10.39 | **0.00** | [1.10, 1.62] |  |
|  | Video games | 0.14 | 0.04 | 3.47 | **0.00** | [0.06, 0.21] |  |
|  | Tobacco use | -0.04 | 0.03 | -1.31 | 0.19 | [-0.11, 0.02] |  |
|  | Alcohol use | 0.01 | 0.03 | 0.24 | 0.81 | [-0.06, 0.07] |  |
|  |  |  |  |  |  |  |  |
|  |  |  |  |  |  |  | R^2^ = 0.258 |
|  |  |  |  |  |  |  | R^2^_adj._ = 0.248 |
| Working memory | Intercept | -0.50 | 0.14 | -3.49 | **0.00** | [-1.24, -0.77] |  |
|  | Cannabis use at age 11 | 0.03 | 0.04 | 0.84 | 0.40 | [-0.11, 0.05] |  |
|  | Sex | 0.07 | 0.08 | 0.83 | 0.41 | [-0.15, 0.01] |  |
|  | SES (ISEI) | 0.07 | 0.04 | 1.60 | 0.11 | [-0.04, 0.25] |  |
|  | Migration background | 0.29 | 0.08 | 3.82 | **0.00** | [0.03, 0.17] |  |
|  | Edu: Apprenticeship | 0.18 | 0.14 | 1.29 | 0.20 | [0.08, 0.34] |  |
|  | Edu: Vocational | 0.39 | 0.15 | 2.66 | **0.01** | [0.41, 0.88] |  |
|  | Edu: Academic | 0.54 | 0.15 | 3.51 | **0.00** | [0.65, 1.14] |  |
|  | Video games | 0.11 | 0.04 | 2.42 | **0.02** | [1.10, 1.62] |  |
|  | Tobacco use | -0.02 | 0.04 | -0.53 | 0.60 | [0.06, 0.21] |  |
|  | Alcohol use | -0.01 | 0.04 | -0.17 | 0.86 | [-0.11, 0.02] |  |
|  |  |  |  |  |  |  | R^2^ = 0.101 |
|  |  |  |  |  |  |  | R^2^_adj._ = 0.090 |
| Working memory | Intercept | -0.56 | 0.15 | -3.77 | **0.00** | [-0.85, -0.27] |  |
|  | Cannabis use at age 13 | -0.02 | 0.04 | -0.45 | 0.65 | [-0.09, 0.06] |  |
|  | Cannabis use at age 11 | 0.03 | 0.06 | 0.62 | 0.54 | [-0.07, 0.14] |  |
|  | Sex | 0.12 | 0.09 | 1.39 | 0.17 | [-0.05, 0.29] |  |
|  | SES (ISEI) | 0.06 | 0.04 | 1.38 | 0.17 | [-0.03, 0.14] |  |
|  | Migration background | 0.31 | 0.08 | 3.90 | **0.00** | [0.16, 0.47] |  |
|  | Edu: Apprenticeship | 0.22 | 0.15 | 1.52 | 0.13 | [-0.06, 0.51] |  |
|  | Edu: Vocational | 0.39 | 0.15 | 2.55 | **0.01** | [0.09, 0.68] |  |
|  | Edu: Academic | 0.57 | 0.16 | 3.57 | **0.00** | [0.26, 0.88] |  |
|  | Video games | 0.09 | 0.05 | 1.94 | 0.05 | [0.00, 0.18] |  |
|  | Tobacco use | -0.02 | 0.04 | -0.64 | 0.52 | [-0.10, 0.05] |  |
|  | Alcohol use | 0.00 | 0.04 | -0.03 | 0.98 | [-0.08, 0.08] |  |
|  |  |  |  |  |  |  | R^2^ = 0.105 |
|  |  |  |  |  |  |  | R^2^_adj._ = 0.091 |
| Working memory | Intercept | -0.46 | 0.13 | -3.57 | **0.00** | [-0.71, -0.21] |  |
|  | Cannabis use at age 15 | -0.01 | 0.04 | -0.19 | 0.85 | [-0.08, 0.07] |  |
|  | Cannabis use at age 13 | -0.02 | 0.04 | -0.63 | 0.53 | [-0.09, 0.05] |  |
|  | Sex | 0.11 | 0.08 | 1.40 | 0.16 | [-0.04, 0.27] |  |
|  | SES (ISEI) | 0.06 | 0.04 | 1.57 | 0.12 | [-0.02, 0.14] |  |
|  | Migration background | 0.28 | 0.07 | 3.86 | **0.00** | [0.14, 0.43] |  |
|  | Edu: Apprenticeship | 0.12 | 0.13 | 0.96 | 0.33 | [-0.13, 0.37] |  |
|  | Edu: Vocational | 0.34 | 0.13 | 2.54 | **0.01** | [0.08, 0.60] |  |
|  | Edu: Academic | 0.48 | 0.14 | 3.36 | **0.00** | [0.20, 0.75] |  |
|  | Video games | 0.11 | 0.04 | 2.66 | **0.01** | [0.03, 0.19] |  |
|  | Tobacco use | -0.03 | 0.03 | -0.92 | 0.36 | [-0.10, 0.04] |  |
|  | Alcohol use | 0.02 | 0.04 | 0.52 | 0.60 | [-0.05, 0.09] |  |
|  |  |  |  |  |  |  | R^2^ = 0.104 |
|  |  |  |  |  |  |  | R^2^_adj._ = 0.093 |
| Working memory | Intercept | -0.43 | 0.13 | -3.27 | **0.00** | [-0.69, -0.17] |  |
|  | Cannabis use at age 17 | -0.02 | 0.04 | -0.40 | 0.69 | [-0.10, 0.07] |  |
|  | Cannabis use at age 15 | 0.02 | 0.04 | 0.42 | 0.68 | [-0.06, 0.09] |  |
|  | Sex | 0.13 | 0.08 | 1.67 | 0.09 | [-0.02, 0.29] |  |
|  | SES (ISEI) | 0.07 | 0.04 | 1.79 | 0.07 | [-0.01, 0.15] |  |
|  | Migration background | 0.32 | 0.07 | 4.45 | **0.00** | [0.18, 0.46] |  |
|  | Edu: Apprenticeship | 0.06 | 0.13 | 0.43 | 0.67 | [-0.20, 0.31] |  |
|  | Edu: Vocational | 0.33 | 0.14 | 2.46 | **0.01** | [0.07, 0.60] |  |
|  | Edu: Academic | 0.40 | 0.14 | 2.75 | **0.01** | [0.11, 0.68] |  |
|  | Video games | 0.11 | 0.04 | 2.67 | **0.01** | [0.03, 0.19] |  |
|  | Tobacco use | -0.02 | 0.04 | -0.62 | 0.54 | [-0.09, 0.05] |  |
|  | Alcohol use | 0.02 | 0.04 | 0.49 | 0.62 | [-0.05, 0.09] |  |
|  |  |  |  |  |  |  | R^2^ = 0.115 |
|  |  |  |  |  |  |  | R^2^_adj._ = 0.104 |
| Working memory | Intercept | -0.37 | 0.14 | -2.76 | **0.01** | [-0.64, -0.11] |  |
|  | Cannabis use at age 20 | 0.03 | 0.04 | 0.80 | 0.43 | [-0.05, 0.12] |  |
|  | Cannabis use at age 17 | -0.01 | 0.04 | -0.25 | 0.80 | [-0.10, 0.07] |  |
|  | Sex | 0.10 | 0.08 | 1.24 | 0.21 | [-0.06, 0.26] |  |
|  | SES (ISEI) | 0.06 | 0.04 | 1.45 | 0.15 | [-0.02, 0.13] |  |
|  | Migration background | 0.31 | 0.07 | 4.35 | **0.00** | [0.17, 0.46] |  |
|  | Edu: Apprenticeship | 0.01 | 0.13 | 0.05 | 0.96 | [-0.26, 0.27] |  |
|  | Edu: Vocational | 0.26 | 0.14 | 1.88 | 0.06 | [-0.01, 0.54] |  |
|  | Edu: Academic | 0.36 | 0.15 | 2.46 | **0.01** | [0.07, 0.65] |  |
|  | Video games | 0.12 | 0.04 | 2.80 | **0.01** | [0.04, 0.21] |  |
|  | Tobacco use | -0.04 | 0.04 | -1.07 | 0.28 | [-0.11, 0.03] |  |
|  | Alcohol use | 0.04 | 0.04 | 1.06 | 0.29 | [-0.03, 0.11] |  |
|  |  |  |  |  |  |  | R^2^ = 0.120 |
|  |  |  |  |  |  |  | R^2^_adj._ = 0.108 |
| Working memory | Intercept | -0.37 | 0.13 | -2.88 | **0.00** | [-0.63, -0.12] |  |
|  | Cannabis use at age 24 | 0.00 | 0.04 | -0.02 | 0.99 | [-0.09, 0.08] |  |
|  | Cannabis use at age 20 | 0.03 | 0.04 | 0.59 | 0.56 | [-0.06, 0.11] |  |
|  | Sex | 0.05 | 0.08 | 0.62 | 0.53 | [-0.11, 0.20] |  |
|  | SES (ISEI) | 0.06 | 0.04 | 1.44 | 0.15 | [-0.02, 0.13] |  |
|  | Migration background | 0.28 | 0.07 | 3.94 | **0.00** | [0.14, 0.42] |  |
|  | Edu: Apprenticeship | 0.06 | 0.13 | 0.49 | 0.62 | [-0.19, 0.31] |  |
|  | Edu: Vocational | 0.27 | 0.13 | 2.02 | 0.04 | [0.01, 0.53] |  |
|  | Edu: Academic | 0.42 | 0.14 | 2.94 | **0.00** | [0.14, 0.70] |  |
|  | Video games | 0.14 | 0.04 | 3.24 | **0.00** | [0.05, 0.22] |  |
|  | Tobacco use | -0.04 | 0.04 | -1.00 | 0.32 | [-0.10, 0.03] |  |
|  | Alcohol use | 0.03 | 0.04 | 0.97 | 0.33 | [-0.04, 0.11] |  |
|  |  |  |  |  |  |  | R^2^ = 0.108 |
|  |  |  |  |  |  |  | R^2^_adj._ = 0.097 |
| Declarative memory | Intercept | -0.29 | 0.15 | -1.90 | 0.06 |  |  |
|  | Cannabis use at age 11 | 0.03 | 0.04 | 0.68 | 0.50 | [-0.58, 0.01] |  |
|  | Sex | 0.02 | 0.09 | 0.23 | 0.82 | [-0.05, 0.19] |  |
|  | SES (ISEI) | -0.02 | 0.04 | -0.53 | 0.60 | [-0.15, 0.06] |  |
|  | Migration background | 0.06 | 0.08 | 0.78 | 0.43 | [-0.11, 0.22] |  |
|  | Edu: Apprenticeship | 0.20 | 0.15 | 1.32 | 0.19 | [-0.10, 0.49] |  |
|  | Edu: Vocational | 0.17 | 0.16 | 1.08 | 0.28 | [-0.14, 0.47] |  |
|  | Edu: Academic | 0.50 | 0.16 | 3.04 | **0.00** | [0.18, 0.82] |  |
|  | Video games | 0.07 | 0.05 | 1.54 | 0.12 | [-0.02, 0.17] |  |
|  | Tobacco use | -0.06 | 0.04 | -1.62 | 0.10 | [-0.14, 0.01] |  |
|  | Alcohol use | 0.08 | 0.04 | 1.96 | 0.05 | [0.00, 0.16] |  |
|  |  |  |  |  |  |  | R^2^ = 0.048 |
|  |  |  |  |  |  |  | R^2^_adj._ = 0.035 |
| Declarative memory | Intercept | -0.32 | 0.15 | -2.19 | **0.03** | [-0.62, -0.03] |  |
|  | Cannabis use at age 13 | -0.13 | 0.04 | -3.40 | **0.00** | [-0.20, -0.05] |  |
|  | Cannabis use at age 11 | 0.04 | 0.06 | 0.74 | 0.46 | [-0.07, 0.15] |  |
|  | Sex | 0.06 | 0.09 | 0.67 | 0.50 | [-0.11, 0.23] |  |
|  | SES (ISEI) | -0.01 | 0.04 | -0.33 | 0.74 | [-0.10, 0.07] |  |
|  | Migration background | 0.08 | 0.08 | 1.01 | 0.31 | [-0.08, 0.24] |  |
|  | Edu: Apprenticeship | 0.25 | 0.15 | 1.70 | 0.09 | [-0.04, 0.54] |  |
|  | Edu: Vocational | 0.17 | 0.15 | 1.13 | 0.26 | [-0.13, 0.47] |  |
|  | Edu: Academic | 0.54 | 0.16 | 3.38 | **0.00** | [0.23, 0.86] |  |
|  | Video games | 0.07 | 0.05 | 1.46 | 0.14 | [-0.02, 0.16] |  |
|  | Tobacco use | -0.04 | 0.04 | -1.14 | 0.26 | [-0.12, 0.03] |  |
|  | Alcohol use | 0.10 | 0.04 | 2.48 | **0.01** | [0.02, 0.18] |  |
|  |  |  |  |  |  |  | R^2^ = 0.077 |
|  |  |  |  |  |  |  | R^2^_adj._ = 0.063 |
| Declarative memory | Intercept | -0.29 | 0.13 | -2.27 | **0.02** | [-0.54, -0.04] |  |
|  | Cannabis use at age 15 | 0.07 | 0.04 | 1.90 | 0.06 | [0.00, 0.14] |  |
|  | Cannabis use at age 13 | -0.13 | 0.04 | -3.72 | **0.00** | [-0.20, -0.06] |  |
|  | Sex | 0.08 | 0.08 | 0.98 | 0.33 | [-0.08, 0.23] |  |
|  | SES (ISEI) | -0.02 | 0.04 | -0.50 | 0.62 | [-0.10, 0.06] |  |
|  | Migration background | 0.00 | 0.07 | -0.01 | 0.99 | [-0.14, 0.14] |  |
|  | Edu: Apprenticeship | 0.21 | 0.12 | 1.67 | 0.10 | [-0.04, 0.45] |  |
|  | Edu: Vocational | 0.22 | 0.13 | 1.70 | 0.09 | [-0.03, 0.48] |  |
|  | Edu: Academic | 0.53 | 0.14 | 3.78 | **0.00** | [0.25, 0.80] |  |
|  | Video games | 0.08 | 0.04 | 1.99 | 0.05 | [0.00, 0.16] |  |
|  | Tobacco use | -0.07 | 0.03 | -2.00 | 0.05 | [-0.14, 0.00] |  |
|  | Alcohol use | 0.10 | 0.04 | 2.77 | **0.01** | [0.03, 0.17] |  |
|  |  |  |  |  |  |  | R^2^ = 0.077 |
|  |  |  |  |  |  |  | R^2^_adj._ = 0.065 |
| Declarative memory | Intercept | -0.16 | 0.13 | -1.22 | 0.22 | [-0.42, 0.10] |  |
|  | Cannabis use at age 17 | -0.09 | 0.04 | -2.21 | **0.03** | [-0.17, -0.01] |  |
|  | Cannabis use at age 15 | 0.10 | 0.04 | 2.43 | **0.02** | [0.02, 0.17] |  |
|  | Sex | 0.04 | 0.08 | 0.46 | 0.65 | [-0.12, 0.19] |  |
|  | SES (ISEI) | -0.01 | 0.04 | -0.34 | 0.73 | [-0.09, 0.06] |  |
|  | Migration background | 0.01 | 0.07 | 0.18 | 0.86 | [-0.13, 0.15] |  |
|  | Edu: Apprenticeship | 0.07 | 0.13 | 0.56 | 0.58 | [-0.18, 0.33] |  |
|  | Edu: Vocational | 0.11 | 0.14 | 0.84 | 0.40 | [-0.15, 0.38] |  |
|  | Edu: Academic | 0.42 | 0.14 | 2.91 | **0.00** | [0.14, 0.70] |  |
|  | Video games | 0.08 | 0.04 | 1.85 | 0.06 | [0.00, 0.16] |  |
|  | Tobacco use | -0.06 | 0.04 | -1.61 | 0.11 | [-0.13, 0.01] |  |
|  | Alcohol use | 0.07 | 0.04 | 1.80 | 0.07 | [-0.01, 0.14] |  |
|  |  |  |  |  |  |  | R^2^ = 0.052 |
|  |  |  |  |  |  |  | R^2^_adj._ = 0.040 |
| Declarative memory | Intercept | -0.09 | 0.14 | -0.69 | 0.49 | [-0.36, 0.17] |  |
|  | Cannabis use at age 20 | -0.02 | 0.04 | -0.40 | 0.69 | [-0.10, 0.07] |  |
|  | Cannabis use at age 17 | -0.03 | 0.04 | -0.72 | 0.47 | [-0.12, 0.05] |  |
|  | Sex | -0.04 | 0.08 | -0.52 | 0.60 | [-0.20, 0.12] |  |
|  | SES (ISEI) | -0.02 | 0.04 | -0.42 | 0.67 | [-0.09, 0.06] |  |
|  | Migration background | 0.04 | 0.07 | 0.50 | 0.62 | [-0.11, 0.18] |  |
|  | Edu: Apprenticeship | 0.02 | 0.14 | 0.15 | 0.88 | [-0.25, 0.29] |  |
|  | Edu: Vocational | 0.08 | 0.14 | 0.60 | 0.55 | [-0.19, 0.36] |  |
|  | Edu: Academic | 0.37 | 0.15 | 2.53 | **0.01** | [0.08, 0.67] |  |
|  | Video games | 0.14 | 0.04 | 3.20 | **0.00** | [0.05, 0.23] |  |
|  | Tobacco use | -0.07 | 0.04 | -1.79 | 0.07 | [-0.14, 0.01] |  |
|  | Alcohol use | 0.08 | 0.04 | 2.17 | **0.03** | [0.01, 0.16] |  |
|  |  |  |  |  |  |  | R^2^ = 0.056 |
|  |  |  |  |  |  |  | R^2^_adj._ = 0.044 |
| Declarative memory | Intercept | -0.17 | 0.13 | -1.30 | 0.20 | [-0.43, 0.09] |  |
|  | Cannabis use at age 24 | -0.08 | 0.04 | -1.77 | 0.08 | [-0.17, 0.01] |  |
|  | Cannabis use at age 20 | 0.04 | 0.05 | 0.88 | 0.38 | [-0.05, 0.13] |  |
|  | Sex | -0.03 | 0.08 | -0.44 | 0.66 | [-0.19, 0.12] |  |
|  | SES (ISEI) | -0.02 | 0.04 | -0.49 | 0.62 | [-0.10, 0.06] |  |
|  | Migration background | 0.02 | 0.07 | 0.30 | 0.77 | [-0.12, 0.16] |  |
|  | Edu: Apprenticeship | 0.11 | 0.13 | 0.81 | 0.42 | [-0.15, 0.36] |  |
|  | Edu: Vocational | 0.17 | 0.14 | 1.25 | 0.21 | [-0.10, 0.44] |  |
|  | Edu: Academic | 0.43 | 0.14 | 2.96 | **0.00** | [0.14, 0.71] |  |
|  | Video games | 0.14 | 0.04 | 3.28 | **0.00** | [0.06, 0.23] |  |
|  | Tobacco use | -0.07 | 0.04 | -1.94 | 0.05 | [-0.14, 0.00] |  |
|  | Alcohol use | 0.09 | 0.04 | 2.43 | **0.02** | [0.02, 0.16] |  |
|  |  |  |  |  |  |  | R^2^ = 0.056 |
|  |  |  |  |  |  |  | R^2^_adj._ = 0.044 |
| Total cognitive index | Intercept | -0.63 | 0.10 | -6.40 | **0.00** | [-0.83, -0.44] |  |
|  | Cannabis use at age 11 | 0.02 | 0.03 | 0.83 | 0.40 | [-0.03, 0.08] |  |
|  | Sex | 0.08 | 0.06 | 1.44 | 0.15 | [-0.03, 0.20] |  |
|  | SES (ISEI) | 0.04 | 0.03 | 1.44 | 0.15 | [-0.01, 0.10] |  |
|  | Migration background | 0.19 | 0.05 | 3.52 | **0.00** | [0.08, 0.29] |  |
|  | Edu: Apprenticeship | 0.37 | 0.10 | 3.75 | **0.00** | [0.17, 0.56] |  |
|  | Edu: Vocational | 0.50 | 0.10 | 4.90 | **0.00** | [0.30, 0.70] |  |
|  | Edu: Academic | 0.82 | 0.11 | 7.61 | **0.00** | [0.60, 1.03] |  |
|  | Video games | 0.09 | 0.03 | 2.85 | **0.00** | [0.03, 0.15] |  |
|  | Tobacco use | -0.05 | 0.03 | -2.13 | **0.03** | [-0.10, 0.00] |  |
|  | Alcohol use | 0.03 | 0.03 | 1.18 | 0.24 | [-0.02, 0.08] |  |
|  |  |  |  |  |  |  | R^2^ = 0.206 |
|  |  |  |  |  |  |  | R^2^_adj._ = 0.196 |
| Total cognitive index | Intercept | -0.67 | 0.10 | -6.75 | **0.00** | [-0.83, -0.44] |  |
|  | Cannabis use at age 13 | -0.06 | 0.03 | -2.19 | **0.03** | [-0.03, 0.08] |  |
|  | Cannabis use at age | 0.03 | 0.04 | 0.88 | 0.38 | [-0.03, 0.20] |  |
|  | Sex | 0.12 | 0.06 | 2.12 | **0.03** | [-0.01, 0.10] |  |
|  | SES (ISEI) | 0.05 | 0.03 | 1.69 | 0.09 | [0.08, 0.29] |  |
|  | Migration background | 0.20 | 0.05 | 3.62 | **0.00** | [0.17, 0.56] |  |
|  | Edu: Apprenticeship | 0.40 | 0.10 | 4.13 | **0.00** | [0.30, 0.70] |  |
|  | Edu: Vocational | 0.50 | 0.10 | 4.86 | **0.00** | [0.60, 1.03] |  |
|  | Edu: Academic | 0.85 | 0.11 | 7.83 | **0.00** | [0.03, 0.15] |  |
|  | Video games | 0.08 | 0.03 | 2.51 | **0.01** | [-0.10, 0.00] |  |
|  | Tobacco use | -0.05 | 0.03 | -1.91 | 0.06 | [-0.02, 0.08] |  |
|  | Alcohol use | 0.04 | 0.03 | 1.61 | 0.11 | [-0.83, -0.44] |  |
|  |  |  |  |  |  |  | R^2^ = 0.235 |
|  |  |  |  |  |  |  | R^2^_adj._ = 0.223 |
| Total cognitive index | Intercept | -0.60 | 0.09 | -6.86 | **0.00** | [-0.77, -0.43] |  |
|  | Cannabis use at age 15 | 0.03 | 0.02 | 1.04 | 0.30 | [-0.02, 0.08] |  |
|  | Cannabis use at age 13 | -0.06 | 0.02 | -2.59 | **0.01** | [-0.11, -0.02] |  |
|  | Sex | 0.11 | 0.05 | 2.02 | **0.04** | [0.00, 0.21] |  |
|  | SES (ISEI) | 0.05 | 0.03 | 1.72 | 0.09 | [-0.01, 0.10] |  |
|  | Migration background | 0.15 | 0.05 | 3.12 | **0.00** | [0.06, 0.25] |  |
|  | Edu: Apprenticeship | 0.33 | 0.09 | 3.92 | **0.00** | [0.17, 0.50] |  |
|  | Edu: Vocational | 0.49 | 0.09 | 5.41 | **0.00** | [0.31, 0.66] |  |
|  | Edu: Academic | 0.80 | 0.10 | 8.35 | **0.00** | [0.61, 0.99] |  |
|  | Video games | 0.11 | 0.03 | 3.78 | **0.00** | [0.05, 0.16] |  |
|  | Tobacco use | -0.06 | 0.02 | -2.36 | **0.02** | [-0.10, -0.01] |  |
|  | Alcohol use | 0.04 | 0.02 | 1.79 | 0.07 | [0.00, 0.09] |  |
|  |  |  |  |  |  |  | R^2^ = 0.230 |
|  |  |  |  |  |  |  | R^2^_adj._ = 0.220 |
| Total cognitive index | Intercept | -0.51 | 0.09 | -5.73 | **0.00** | [-0.69, -0.34] |  |
|  | Cannabis use at age 17 | -0.05 | 0.03 | -1.86 | 0.06 | [-0.11, 0.00] |  |
|  | Cannabis use at age 15 | 0.05 | 0.03 | 1.69 | 0.09 | [-0.01, 0.10] |  |
|  | Sex | 0.10 | 0.05 | 1.81 | 0.07 | [-0.01, 0.20] |  |
|  | SES (ISEI) | 0.05 | 0.03 | 1.87 | 0.06 | [0.00, 0.10] |  |
|  | Migration background | 0.18 | 0.05 | 3.71 | **0.00** | [0.08, 0.27] |  |
|  | Edu: Apprenticeship | 0.22 | 0.09 | 2.51 | **0.01** | [0.05, 0.39] |  |
|  | Edu: Vocational | 0.42 | 0.09 | 4.55 | **0.00** | [0.24, 0.60] |  |
|  | Edu: Academic | 0.70 | 0.10 | 7.16 | **0.00** | [0.50, 0.89] |  |
|  | Video games | 0.10 | 0.03 | 3.60 | **0.00** | [0.05, 0.16] |  |
|  | Tobacco use | -0.05 | 0.02 | -1.93 | 0.05 | [-0.09, 0.00] |  |
|  | Alcohol use | 0.03 | 0.02 | 1.11 | 0.27 | [-0.02, 0.08] |  |
|  |  |  |  |  |  |  | R^2^ = 0.213 |
|  |  |  |  |  |  |  | R^2^_adj._ = 0.203 |
| Total cognitive index | Intercept | -0.48 | 0.09 | -5.16 | **0.00** | [-0.66, -0.29] |  |
|  | Cannabis use at age 20 | -0.03 | 0.03 | -0.90 | 0.37 | [-0.08, 0.03] |  |
|  | Cannabis use at age 17 | -0.01 | 0.03 | -0.35 | 0.73 | [-0.07, 0.05] |  |
|  | Sex | 0.06 | 0.05 | 1.07 | 0.29 | [-0.05, 0.17] |  |
|  | SES (ISEI) | 0.05 | 0.03 | 1.77 | 0.08 | [-0.01, 0.10] |  |
|  | Migration background | 0.19 | 0.05 | 3.80 | **0.00** | [0.09, 0.28] |  |
|  | Edu: Apprenticeship | 0.20 | 0.09 | 2.14 | **0.03** | [0.02, 0.38] |  |
|  | Edu: Vocational | 0.40 | 0.10 | 4.14 | **0.00** | [0.21, 0.58] |  |
|  | Edu: Academic | 0.68 | 0.10 | 6.76 | **0.00** | [0.48, 0.87] |  |
|  | Video games | 0.13 | 0.03 | 4.27 | **0.00** | [0.07, 0.19] |  |
|  | Tobacco use | -0.05 | 0.02 | -2.08 | **0.04** | [-0.10, 0.00] |  |
|  | Alcohol use | 0.04 | 0.03 | 1.72 | 0.09 | [-0.01, 0.09] |  |
|  |  |  |  |  |  |  | R^2^ = 0.221 |
|  |  |  |  |  |  |  | R^2^_adj._ = 0.211 |
| Total cognitive index | Intercept | -0.52 | 0.09 | -5.87 | **0.00** | [-0.69, -0.34] |  |
|  | Cannabis use at age 24 | -0.04 | 0.03 | -1.20 | 0.23 | [-0.09, 0.02] |  |
|  | Cannabis use at age 20 | 0.00 | 0.03 | -0.06 | 0.95 | [-0.06, 0.06] |  |
|  | Sex | 0.04 | 0.05 | 0.74 | 0.46 | [-0.07, 0.14] |  |
|  | SES (ISEI) | 0.05 | 0.03 | 1.75 | 0.08 | [-0.01, 0.10] |  |
|  | Migration background | 0.17 | 0.05 | 3.55 | **0.00** | [0.08, 0.27] |  |
|  | Edu: Apprenticeship | 0.27 | 0.09 | 3.09 | **0.00** | [0.10, 0.44] |  |
|  | Edu: Vocational | 0.45 | 0.09 | 4.89 | **0.00** | [0.27, 0.63] |  |
|  | Edu: Academic | 0.74 | 0.10 | 7.63 | **0.00** | [0.55, 0.93] |  |
|  | Video games | 0.14 | 0.03 | 4.77 | **0.00** | [0.08, 0.20] |  |
|  | Tobacco use | -0.05 | 0.02 | -2.04 | **0.04** | [-0.10, 0.00] |  |
|  | Alcohol use | 0.04 | 0.02 | 1.72 | 0.08 | [-0.01, 0.09] |  |
|  |  |  |  |  |  |  | R^2^ = 0.218 |
|  |  |  |  |  |  |  | R^2^_adj._ = 0.208 |

# **Table S3.** Quadratic regressions with sociodemographic and behavioral variables as covariates for each time point.

|  | Term | Estimate | *SE* | *t* | *p* | 95% CI | Fit |
| --- | --- | --- | --- | --- | --- | --- | --- |
| Sustained attention | Intercept | -1.11 | 0.13 | -8.43 | **0.00** | [-1.37, -0.85] |  |
|  | Cannabis use at age 11 | 0.01 | 0.04 | 0.20 | 0.84 | [-0.06, 0.08] |  |
|  | Quadratic term | *NA* | *NA* | *NA* | *NA* | *NA* |  |
|  | Sex | 0.16 | 0.08 | 2.02 | **0.04** | [0.00, 0.31] |  |
|  | SES (ISEI) | 0.07 | 0.04 | 1.97 | 0.05 | [0.00, 0.15] |  |
|  | Migration background | 0.21 | 0.07 | 2.99 | **0.00** | [0.07, 0.35] |  |
|  | Edu: Apprenticeship | 0.73 | 0.13 | 5.57 | **0.00** | [0.47, 0.98] |  |
|  | Edu: Vocational | 0.95 | 0.14 | 7.00 | **0.00** | [0.69, 1.22] |  |
|  | Edu: Academic | 1.41 | 0.14 | 9.82 | **0.00** | [1.13, 1.69] |  |
|  | Video games | 0.08 | 0.04 | 2.04 | **0.04** | [0.00, 0.17] |  |
|  | Tobacco use | -0.08 | 0.03 | -2.44 | **0.01** | [-0.15, -0.02] |  |
|  | Alcohol use | 0.02 | 0.04 | 0.50 | 0.62 | [-0.05, 0.09] |  |
|  |  |  |  |  |  |  | R^2^ = 0.248 |
|  |  |  |  |  |  |  | R^2^_adj._ = 0.238 |
| Sustained attention | Intercept | -1.13 | 0.14 | -8.18 | **0.00** | [-1.41, -0.86] |  |
|  | Cannabis use at age 13 | 0.02 | 0.08 | 0.25 | 0.80 | [-0.14, 0.18] |  |
|  | Quadratic term | -0.01 | 0.02 | -0.58 | 0.56 | [-0.04, 0.02] |  |
|  | Cannabis use at age 11 | 0.02 | 0.05 | 0.38 | 0.70 | [-0.08, 0.12] |  |
|  | Sex | 0.19 | 0.08 | 2.31 | **0.02** | [0.03, 0.34] |  |
|  | SES (ISEI) | 0.09 | 0.04 | 2.38 | **0.02** | [0.02, 0.17] |  |
|  | Migration background | 0.20 | 0.07 | 2.74 | **0.01** | [0.06, 0.35] |  |
|  | Edu: Apprenticeship | 0.75 | 0.13 | 5.57 | **0.00** | [0.49, 1.02] |  |
|  | Edu: Vocational | 0.95 | 0.14 | 6.75 | **0.00** | [0.68, 1.23] |  |
|  | Edu: Academic | 1.42 | 0.15 | 9.58 | **0.00** | [1.13, 1.72] |  |
|  | Video games | 0.08 | 0.04 | 1.80 | 0.07 | [-0.01, 0.16] |  |
|  | Tobacco use | -0.08 | 0.04 | -2.33 | **0.02** | [-0.15, -0.01] |  |
|  | Alcohol use | 0.03 | 0.04 | 0.71 | 0.48 | [-0.05, 0.10] |  |
|  |  |  |  |  |  |  | R^2^ = 0.261 |
|  |  |  |  |  |  |  | R^2^_adj._ = 0.249 |
| Sustained attention | Intercept | -1.07 | 0.12 | -8.58 | **0.00** | [-0.04, 0.06] |  |
|  | Cannabis use at age 15 | 0.00 | 0.06 | -0.08 | 0.93 | [-0.11, 0.03] |  |
|  | Quadratic term | 0.01 | 0.03 | 0.36 | 0.72 | [0.00, 0.29] |  |
|  | Cannabis use at age 13 | -0.04 | 0.03 | -1.11 | 0.27 | [0.02, 0.17] |  |
|  | Sex | 0.15 | 0.07 | 2.00 | 0.05 | [0.06, 0.33] |  |
|  | SES (ISEI) | 0.10 | 0.04 | 2.55 | **0.01** | [0.45, 0.91] |  |
|  | Migration background | 0.19 | 0.07 | 2.85 | **0.00** | [0.67, 1.15] |  |
|  | Edu: Apprenticeship | 0.68 | 0.12 | 5.83 | **0.00** | [1.14, 1.66] |  |
|  | Edu: Vocational | 0.91 | 0.12 | 7.36 | **0.00** | [0.04, 0.20] |  |
|  | Edu: Academic | 1.40 | 0.13 | 10.57 | **0.00** | [-0.13, 0.00] |  |
|  | Video games | 0.12 | 0.04 | 3.12 | **0.00** | [-0.06, 0.08] |  |
|  | Tobacco use | -0.06 | 0.03 | -2.00 | 0.05 | [-0.04, 0.06] |  |
|  | Alcohol use | 0.01 | 0.03 | 0.26 | 0.79 | [-0.11, 0.03] |  |
|  |  |  |  |  |  |  | R^2^ = 0.256 |
|  |  |  |  |  |  |  | R^2^_adj._ = 0.245 |
| Sustained attention | Intercept | -0.90 | 0.13 | -6.95 | **0.00** | [-1.16, -0.65] |  |
|  | Cannabis use at age 17 | -0.01 | 0.05 | -0.26 | 0.80 | [-0.11, 0.09] |  |
|  | Quadratic term | -0.03 | 0.03 | -1.06 | 0.29 | [-0.10, 0.03] |  |
|  | Cannabis use at age 15 | 0.03 | 0.04 | 0.68 | 0.50 | [-0.05, 0.10] |  |
|  | Sex | 0.13 | 0.07 | 1.77 | 0.08 | [-0.01, 0.28] |  |
|  | SES (ISEI) | 0.09 | 0.04 | 2.44 | **0.01** | [0.02, 0.16] |  |
|  | Migration background | 0.20 | 0.07 | 2.93 | **0.00** | [0.06, 0.33] |  |
|  | Edu: Apprenticeship | 0.54 | 0.12 | 4.39 | **0.00** | [0.30, 0.78] |  |
|  | Edu: Vocational | 0.80 | 0.13 | 6.27 | **0.00** | [0.55, 1.05] |  |
|  | Edu: Academic | 1.26 | 0.14 | 9.35 | **0.00** | [1.00, 1.53] |  |
|  | Video games | 0.12 | 0.04 | 2.99 | **0.00** | [0.04, 0.19] |  |
|  | Tobacco use | -0.06 | 0.03 | -1.93 | 0.05 | [-0.13, 0.00] |  |
|  | Alcohol use | 0.00 | 0.03 | -0.04 | 0.97 | [-0.07, 0.07] |  |
|  |  |  |  |  |  |  | R^2^ = 0.238 |
|  |  |  |  |  |  |  | R^2^_adj._ = 0.228 |
| Sustained attention | Intercept | -0.92 | 0.13 | -7.03 | **0.00** | [-1.18, -0.66] |  |
|  | Cannabis use at age 20 | -0.06 | 0.05 | -1.21 | 0.23 | [-0.16, 0.04] |  |
|  | Quadratic term | -0.04 | 0.03 | -1.08 | 0.28 | [-0.10, 0.03] |  |
|  | Cannabis use at age 17 | 0.02 | 0.04 | 0.39 | 0.70 | [-0.06, 0.09] |  |
|  | Sex | 0.12 | 0.08 | 1.62 | 0.11 | [-0.03, 0.27] |  |
|  | SES (ISEI) | 0.10 | 0.04 | 2.60 | **0.01** | [0.02, 0.17] |  |
|  | Migration background | 0.21 | 0.07 | 3.10 | **0.00** | [0.08, 0.34] |  |
|  | Edu: Apprenticeship | 0.56 | 0.13 | 4.48 | **0.00** | [0.32, 0.81] |  |
|  | Edu: Vocational | 0.83 | 0.13 | 6.33 | **0.00** | [0.57, 1.09] |  |
|  | Edu: Academic | 1.28 | 0.14 | 9.33 | **0.00** | [1.01, 1.55] |  |
|  | Video games | 0.12 | 0.04 | 2.94 | **0.00** | [0.04, 0.20] |  |
|  | Tobacco use | -0.05 | 0.03 | -1.59 | 0.11 | [-0.12, 0.01] |  |
|  | Alcohol use | 0.01 | 0.04 | 0.26 | 0.79 | [-0.06, 0.08] |  |
|  |  |  |  |  |  |  | R^2^ = 0.253 |
|  |  |  |  |  |  |  | R^2^_adj._ = 0.242 |
| Sustained attention | Intercept | -0.97 | 0.12 | -7.81 | **0.00** | [-1.21, -0.72] |  |
|  | Cannabis use at age 24 | 0.02 | 0.06 | 0.36 | 0.72 | [-0.10, 0.14] |  |
|  | Quadratic term | -0.03 | 0.03 | -1.11 | 0.27 | [-0.09, 0.03] |  |
|  | Cannabis use at age 20 | -0.07 | 0.04 | -1.71 | 0.09 | [-0.15, 0.01] |  |
|  | Sex | 0.10 | 0.07 | 1.45 | 0.15 | [-0.04, 0.25] |  |
|  | SES (ISEI) | 0.10 | 0.04 | 2.74 | **0.01** | [0.03, 0.17] |  |
|  | Migration background | 0.21 | 0.07 | 3.22 | **0.00** | [0.08, 0.34] |  |
|  | Edu: Apprenticeship | 0.65 | 0.12 | 5.46 | **0.00** | [0.41, 0.88] |  |
|  | Edu: Vocational | 0.89 | 0.12 | 7.20 | **0.00** | [0.65, 1.14] |  |
|  | Edu: Academic | 1.36 | 0.13 | 10.37 | **0.00** | [1.10, 1.62] |  |
|  | Video games | 0.14 | 0.04 | 3.51 | **0.00** | [0.06, 0.22] |  |
|  | Tobacco use | -0.05 | 0.03 | -1.41 | 0.16 | [-0.11, 0.02] |  |
|  | Alcohol use | 0.00 | 0.03 | 0.04 | 0.97 | [-0.06, 0.07] |  |
|  |  |  |  |  |  |  | R^2^ = 0.259 |
|  |  |  |  |  |  |  | R^2^_adj._ = 0.249 |
| Working memory | Intercept | -0.50 | 0.14 | -3.49 | **0.00** | [-0.78, -0.22] |  |
|  | Cannabis use at age 11 | 0.03 | 0.04 | 0.84 | 0.40 | [-0.04, 0.11] |  |
|  | Quadratic term | *NA* | *NA* | *NA* | *NA* | *NA* |  |
|  | Sex | 0.07 | 0.08 | 0.83 | 0.41 | [-0.09, 0.23] |  |
|  | SES (ISEI) | 0.07 | 0.04 | 1.60 | 0.11 | [-0.01, 0.15] |  |
|  | Migration background | 0.29 | 0.08 | 3.82 | **0.00** | [0.14, 0.44] |  |
|  | Edu: Apprenticeship | 0.18 | 0.14 | 1.29 | 0.20 | [-0.09, 0.46] |  |
|  | Edu: Vocational | 0.39 | 0.15 | 2.66 | **0.01** | [0.10, 0.68] |  |
|  | Edu: Academic | 0.54 | 0.15 | 3.51 | **0.00** | [0.24, 0.85] |  |
|  | Video games | 0.11 | 0.04 | 2.42 | **0.02** | [0.02, 0.20] |  |
|  | Tobacco use | -0.02 | 0.04 | -0.53 | 0.60 | [-0.09, 0.05] |  |
|  | Alcohol use | -0.01 | 0.04 | -0.17 | 0.86 | [-0.08, 0.07] |  |
|  |  |  |  |  |  |  | R^2^ = 0.101 |
|  |  |  |  |  |  |  | R^2^_adj._ = 0.090 |
| Working memory | Intercept | -0.55 | 0.15 | -3.70 | **0.00** | [-0.84, -0.26] |  |
|  | Cannabis use at age 13 | 0.01 | 0.09 | 0.11 | 0.91 | [-0.17, 0.19] |  |
|  | Quadratic term | -0.01 | 0.02 | -0.33 | 0.74 | [-0.04, 0.03] |  |
|  | Cannabis use at age 11 | 0.03 | 0.06 | 0.58 | 0.56 | [-0.08, 0.14] |  |
|  | Sex | 0.12 | 0.09 | 1.37 | 0.17 | [-0.05, 0.29] |  |
|  | SES (ISEI) | 0.06 | 0.04 | 1.36 | 0.17 | [-0.03, 0.14] |  |
|  | Migration background | 0.31 | 0.08 | 3.90 | **0.00** | [0.16, 0.47] |  |
|  | Edu: Apprenticeship | 0.22 | 0.15 | 1.52 | 0.13 | [-0.06, 0.51] |  |
|  | Edu: Vocational | 0.39 | 0.15 | 2.55 | **0.01** | [0.09, 0.69] |  |
|  | Edu: Academic | 0.57 | 0.16 | 3.58 | **0.00** | [0.26, 0.89] |  |
|  | Video games | 0.09 | 0.05 | 1.94 | 0.05 | [0.00, 0.18] |  |
|  | Tobacco use | -0.02 | 0.04 | -0.64 | 0.52 | [-0.10, 0.05] |  |
|  | Alcohol use | 0.00 | 0.04 | -0.06 | 0.96 | [-0.08, 0.08] |  |
|  |  |  |  |  |  |  | R^2^ = 0.105 |
|  |  |  |  |  |  |  | R^2^_adj._ = 0.090 |
| Working memory | Intercept | -0.48 | 0.13 | -3.63 | **0.00** | [-0.75, -0.22] |  |
|  | Cannabis use at age 15 | -0.04 | 0.06 | -0.67 | 0.50 | [-0.17, 0.08] |  |
|  | Quadratic term | 0.02 | 0.03 | 0.68 | 0.49 | [-0.04, 0.08] |  |
|  | Cannabis use at age 13 | -0.03 | 0.04 | -0.72 | 0.47 | [-0.10, 0.05] |  |
|  | Sex | 0.11 | 0.08 | 1.41 | 0.16 | [-0.04, 0.27] |  |
|  | SES (ISEI) | 0.06 | 0.04 | 1.59 | 0.11 | [-0.01, 0.14] |  |
|  | Migration background | 0.28 | 0.07 | 3.87 | **0.00** | [0.14, 0.43] |  |
|  | Edu: Apprenticeship | 0.12 | 0.13 | 0.98 | 0.33 | [-0.12, 0.37] |  |
|  | Edu: Vocational | 0.34 | 0.13 | 2.57 | **0.01** | [0.08, 0.60] |  |
|  | Edu: Academic | 0.48 | 0.14 | 3.38 | **0.00** | [0.20, 0.76] |  |
|  | Video games | 0.11 | 0.04 | 2.66 | **0.01** | [0.03, 0.19] |  |
|  | Tobacco use | -0.03 | 0.03 | -0.86 | 0.39 | [-0.10, 0.04] |  |
|  | Alcohol use | 0.02 | 0.04 | 0.51 | 0.61 | [-0.05, 0.09] |  |
|  |  |  |  |  |  |  | R^2^ = 0.105 |
|  |  |  |  |  |  |  | R^2^_adj._ = 0.092 |
| Working memory | Intercept | -0.42 | 0.14 | -3.06 | **0.00** | [-0.70, -0.15] |  |
|  | Cannabis use at age 17 | -0.01 | 0.05 | -0.18 | 0.86 | [-0.12, 0.10] |  |
|  | Quadratic term | -0.01 | 0.04 | -0.19 | 0.85 | [-0.08, 0.06] |  |
|  | Cannabis use at age 15 | 0.02 | 0.04 | 0.43 | 0.67 | [-0.06, 0.09] |  |
|  | Sex | 0.13 | 0.08 | 1.68 | 0.09 | [-0.02, 0.29] |  |
|  | SES (ISEI) | 0.07 | 0.04 | 1.79 | 0.07 | [-0.01, 0.15] |  |
|  | Migration background | 0.32 | 0.07 | 4.41 | **0.00** | [0.18, 0.46] |  |
|  | Edu: Apprenticeship | 0.06 | 0.13 | 0.43 | 0.67 | [-0.20, 0.31] |  |
|  | Edu: Vocational | 0.33 | 0.14 | 2.45 | **0.01** | [0.07, 0.60] |  |
|  | Edu: Academic | 0.39 | 0.14 | 2.72 | **0.01** | [0.11, 0.68] |  |
|  | Video games | 0.11 | 0.04 | 2.67 | **0.01** | [0.03, 0.19] |  |
|  | Tobacco use | -0.02 | 0.04 | -0.63 | 0.53 | [-0.09, 0.05] |  |
|  | Alcohol use | 0.02 | 0.04 | 0.47 | 0.64 | [-0.05, 0.09] |  |
|  |  |  |  |  |  |  | R^2^ = 0.115 |
|  |  |  |  |  |  |  | R^2^_adj._ = 0.103 |
| Working memory | Intercept | -0.37 | 0.14 | -2.67 | **0.01** | [-0.65, -0.10] |  |
|  | Cannabis use at age 20 | 0.03 | 0.06 | 0.60 | 0.55 | [-0.08, 0.14] |  |
|  | Quadratic term | 0.00 | 0.04 | 0.02 | 0.98 | [-0.07, 0.07] |  |
|  | Cannabis use at age 17 | -0.01 | 0.04 | -0.25 | 0.80 | [-0.10, 0.07] |  |
|  | Sex | 0.10 | 0.08 | 1.24 | 0.22 | [-0.06, 0.26] |  |
|  | SES (ISEI) | 0.06 | 0.04 | 1.45 | 0.15 | [-0.02, 0.13] |  |
|  | Migration background | 0.31 | 0.07 | 4.35 | **0.00** | [0.17, 0.46] |  |
|  | Edu: Apprenticeship | 0.01 | 0.13 | 0.05 | 0.96 | [-0.26, 0.27] |  |
|  | Edu: Vocational | 0.26 | 0.14 | 1.88 | 0.06 | [-0.01, 0.54] |  |
|  | Edu: Academic | 0.36 | 0.15 | 2.46 | **0.01** | [0.07, 0.65] |  |
|  | Video games | 0.12 | 0.04 | 2.80 | **0.01** | [0.04, 0.21] |  |
|  | Tobacco use | -0.04 | 0.04 | -1.07 | 0.29 | [-0.11, 0.03] |  |
|  | Alcohol use | 0.04 | 0.04 | 1.05 | 0.30 | [-0.03, 0.11] |  |
|  |  |  |  |  |  |  | R^2^ = 0.120 |
|  |  |  |  |  |  |  | R^2^_adj._ = 0.107 |
| Working memory | Intercept | -0.43 | 0.13 | -3.22 | **0.00** | [-0.70, -0.17] |  |
|  | Cannabis use at age 24 | -0.08 | 0.06 | -1.25 | 0.21 | [-0.21, 0.05] |  |
|  | Quadratic term | 0.06 | 0.03 | 1.68 | 0.09 | [-0.01, 0.12] |  |
|  | Cannabis use at age 20 | 0.02 | 0.04 | 0.48 | 0.63 | [-0.07, 0.11] |  |
|  | Sex | 0.05 | 0.08 | 0.62 | 0.53 | [-0.10, 0.20] |  |
|  | SES (ISEI) | 0.06 | 0.04 | 1.45 | 0.15 | [-0.02, 0.13] |  |
|  | Migration background | 0.28 | 0.07 | 3.95 | **0.00** | [0.14, 0.42] |  |
|  | Edu: Apprenticeship | 0.06 | 0.13 | 0.49 | 0.63 | [-0.19, 0.31] |  |
|  | Edu: Vocational | 0.28 | 0.13 | 2.07 | **0.04** | [0.01, 0.54] |  |
|  | Edu: Academic | 0.42 | 0.14 | 2.97 | **0.00** | [0.14, 0.70] |  |
|  | Video games | 0.14 | 0.04 | 3.16 | **0.00** | [0.05, 0.22] |  |
|  | Tobacco use | -0.03 | 0.04 | -0.84 | 0.40 | [-0.10, 0.04] |  |
|  | Alcohol use | 0.05 | 0.04 | 1.24 | 0.21 | [-0.03, 0.12] |  |
|  |  |  |  |  |  |  | R^2^ = 0.110 |
|  |  |  |  |  |  |  | R^2^_adj._ = 0.098 |
| Declarative memory | Intercept | -0.29 | 0.15 | -1.90 | 0.06 | [-0.58, 0.01] |  |
|  | Cannabis use at age 11 | 0.03 | 0.04 | 0.68 | 0.50 | [-0.05, 0.11] |  |
|  | Quadratic term | *NA* | *NA* | *NA* | *NA* | *NA* |  |
|  | Sex | 0.02 | 0.09 | 0.23 | 0.82 | [-0.15, 0.19] |  |
|  | SES (ISEI) | -0.02 | 0.04 | -0.53 | 0.60 | [-0.11, 0.06] |  |
|  | Migration background | 0.06 | 0.08 | 0.78 | 0.43 | [-0.10, 0.22] |  |
|  | Edu: Apprenticeship | 0.20 | 0.15 | 1.32 | 0.19 | [-0.10, 0.49] |  |
|  | Edu: Vocational | 0.17 | 0.16 | 1.08 | 0.28 | [-0.14, 0.47] |  |
|  | Edu: Academic | 0.50 | 0.16 | 3.04 | **0.00** | [0.18, 0.82] |  |
|  | Video games | 0.07 | 0.05 | 1.54 | 0.12 | [-0.02, 0.17] |  |
|  | Tobacco use | -0.06 | 0.04 | -1.62 | 0.10 | [-0.14, 0.01] |  |
|  | Alcohol use | 0.08 | 0.04 | 1.96 | 0.05 | [0.00, 0.16] |  |
|  |  |  |  |  |  |  | R^2^ = 0.048 |
|  |  |  |  |  |  |  | R^2^_adj._ = 0.035 |
| Declarative memory | Intercept | -0.34 | 0.15 | -2.27 | **0.02** | [-0.63, -0.05] |  |
|  | Cannabis use at age 13 | -0.20 | 0.09 | -2.21 | **0.03** | [-0.37, -0.02] |  |
|  | Quadratic term | 0.02 | 0.02 | 0.85 | 0.40 | [-0.02, 0.05] |  |
|  | Cannabis use at age 11 | 0.05 | 0.06 | 0.82 | 0.41 | [-0.06, 0.16] |  |
|  | Sex | 0.06 | 0.09 | 0.71 | 0.48 | [-0.11, 0.23] |  |
|  | SES (ISEI) | -0.01 | 0.04 | -0.29 | 0.77 | [-0.10, 0.07] |  |
|  | Migration background | 0.08 | 0.08 | 1.00 | 0.32 | [-0.08, 0.24] |  |
|  | Edu: Apprenticeship | 0.25 | 0.15 | 1.71 | 0.09 | [-0.04, 0.54] |  |
|  | Edu: Vocational | 0.17 | 0.15 | 1.11 | 0.27 | [-0.13, 0.47] |  |
|  | Edu: Academic | 0.54 | 0.16 | 3.36 | **0.00** | [0.22, 0.86] |  |
|  | Video games | 0.07 | 0.05 | 1.46 | 0.14 | [-0.02, 0.16] |  |
|  | Tobacco use | -0.04 | 0.04 | -1.14 | 0.26 | [-0.12, 0.03] |  |
|  | Alcohol use | 0.10 | 0.04 | 2.54 | **0.01** | [0.02, 0.18] |  |
|  |  |  |  |  |  |  | R^2^ = 0.078 |
|  |  |  |  |  |  |  | R^2^_adj._ = 0.063 |
| Declarative memory | Intercept | -0.20 | 0.13 | -1.53 | 0.13 | [-0.87, -0.48] |  |
|  | Cannabis use at age 15 | 0.20 | 0.06 | 3.17 | **0.00** | [-0.17, 0.06] |  |
|  | Quadratic term | -0.07 | 0.03 | -2.53 | **0.01** | [-0.02, 0.02] |  |
|  | Cannabis use at age 13 | -0.12 | 0.04 | -3.30 | **0.00** | [-0.04, 0.11] |  |
|  | Sex | 0.07 | 0.08 | 0.92 | 0.36 | [0.01, 0.24] |  |
|  | SES (ISEI) | -0.02 | 0.04 | -0.58 | 0.56 | [-0.01, 0.11] |  |
|  | Migration background | 0.00 | 0.07 | -0.04 | 0.97 | [0.09, 0.30] |  |
|  | Edu: Apprenticeship | 0.20 | 0.12 | 1.60 | 0.11 | [0.21, 0.60] |  |
|  | Edu: Vocational | 0.21 | 0.13 | 1.60 | 0.11 | [0.30, 0.70] |  |
|  | Edu: Academic | 0.52 | 0.14 | 3.71 | **0.00** | [0.63, 1.06] |  |
|  | Video games | 0.08 | 0.04 | 2.01 | 0.05 | [0.02, 0.14] |  |
|  | Tobacco use | -0.08 | 0.03 | -2.20 | **0.03** | [-0.10, 0.00] |  |
|  | Alcohol use | 0.10 | 0.04 | 2.81 | **0.00** | [-0.01, 0.10] |  |
|  |  |  |  |  |  |  | R^2^ = 0.084 |
|  |  |  |  |  |  |  | R^2^_adj._ = 0.071 |
| Declarative memory | Intercept | -0.11 | 0.14 | -0.77 | 0.44 | [-0.38, 0.16] |  |
|  | Cannabis use at age 17 | -0.05 | 0.05 | -0.84 | 0.40 | [-0.15, 0.06] |  |
|  | Quadratic term | -0.05 | 0.03 | -1.29 | 0.20 | [-0.11, 0.02] |  |
|  | Cannabis use at age 15 | 0.10 | 0.04 | 2.52 | **0.01** | [0.02, 0.18] |  |
|  | Sex | 0.04 | 0.08 | 0.56 | 0.58 | [-0.11, 0.20] |  |
|  | SES (ISEI) | -0.01 | 0.04 | -0.34 | 0.73 | [-0.09, 0.06] |  |
|  | Migration background | 0.00 | 0.07 | 0.05 | 0.96 | [-0.14, 0.14] |  |
|  | Edu: Apprenticeship | 0.07 | 0.13 | 0.52 | 0.60 | [-0.05, 0.44] |  |
|  | Edu: Vocational | 0.10 | 0.14 | 0.77 | 0.44 | [-0.05, 0.47] |  |
|  | Edu: Academic | 0.40 | 0.14 | 2.80 | **0.01** | [0.24, 0.79] |  |
|  | Video games | 0.08 | 0.04 | 1.87 | 0.06 | [0.00, 0.16] |  |
|  | Tobacco use | -0.06 | 0.04 | -1.72 | 0.09 | [-0.13, 0.01] |  |
|  | Alcohol use | 0.06 | 0.04 | 1.68 | 0.09 | [-0.01, 0.03] |  |
|  |  |  |  |  |  |  | R^2^ = 0.054 |
|  |  |  |  |  |  |  | R^2^_adj._ = 0.041 |
| Declarative memory | Intercept | -0.03 | 0.14 | -0.18 | 0.86 | [-0.30, 0.25] |  |
|  | Cannabis use at age 20 | 0.05 | 0.06 | 0.89 | 0.37 | [-0.06, 0.16] |  |
|  | Quadratic term | -0.07 | 0.04 | -1.90 | 0.06 | [-0.14, 0.00] |  |
|  | Cannabis use at age 17 | -0.03 | 0.04 | -0.59 | 0.55 | [-0.11, 0.06] |  |
|  | Sex | -0.03 | 0.08 | -0.41 | 0.68 | [-0.19, 0.13] |  |
|  | SES (ISEI) | -0.02 | 0.04 | -0.51 | 0.61 | [-0.10, 0.06] |  |
|  | Migration background | 0.04 | 0.07 | 0.54 | 0.59 | [-0.10, 0.18] |  |
|  | Edu: Apprenticeship | 0.02 | 0.14 | 0.13 | 0.90 | [-0.25, 0.28] |  |
|  | Edu: Vocational | 0.07 | 0.14 | 0.50 | 0.62 | [-0.21, 0.35] |  |
|  | Edu: Academic | 0.36 | 0.15 | 2.45 | **0.01** | [0.07, 0.65] |  |
|  | Video games | 0.14 | 0.04 | 3.20 | **0.00** | [0.05, 0.23] |  |
|  | Tobacco use | -0.07 | 0.04 | -1.96 | 0.05 | [-0.14, 0.00] |  |
|  | Alcohol use | 0.07 | 0.04 | 1.84 | 0.07 | [0.00, 0.14] |  |
|  |  |  |  |  |  |  | R^2^ = 0.060 |
|  |  |  |  |  |  |  | R^2^_adj._ = 0.047 |
| Declarative memory | Intercept | -0.10 | 0.14 | -0.75 | 0.45 | [-0.37, 0.17] |  |
|  | Cannabis use at age 24 | 0.01 | 0.07 | 0.20 | 0.85 | [-0.12, 0.14] |  |
|  | Quadratic term | -0.06 | 0.03 | -1.88 | 0.06 | [-0.13, 0.00] |  |
|  | Cannabis use at age 20 | 0.05 | 0.05 | 1.00 | 0.32 | [-0.04, 0.13] |  |
|  | Sex | -0.04 | 0.08 | -0.44 | 0.66 | [-0.19, 0.12] |  |
|  | SES (ISEI) | -0.02 | 0.04 | -0.50 | 0.62 | [-0.10, 0.06] |  |
|  | Migration background | 0.02 | 0.07 | 0.29 | 0.77 | [-0.12, 0.16] |  |
|  | Edu: Apprenticeship | 0.11 | 0.13 | 0.82 | 0.41 | [-0.15, 0.36] |  |
|  | Edu: Vocational | 0.16 | 0.14 | 1.19 | 0.23 | [-0.11, 0.43] |  |
|  | Edu: Academic | 0.42 | 0.14 | 2.93 | **0.00** | [0.14, 0.71] |  |
|  | Video games | 0.15 | 0.04 | 3.36 | **0.00** | [0.06, 0.23] |  |
|  | Tobacco use | -0.08 | 0.04 | -2.10 | **0.04** | [-0.15, -0.01] |  |
|  | Alcohol use | 0.08 | 0.04 | 2.07 | **0.04** | [0.00, 0.15] |  |
|  |  |  |  |  |  |  | R^2^ = 0.060 |
|  |  |  |  |  |  |  | R^2^_adj._ = 0.047 |
| Total cognitive index | Intercept | -0.63 | 0.10 | -6.40 | **0.00** | [-0.69, -0.34] |  |
|  | Cannabis use at age 11 | 0.02 | 0.03 | 0.83 | 0.40 | [-0.09, 0.02] |  |
|  | Quadratic term | *NA* | *NA* | *NA* | *NA* | *NA* |  |
|  | Sex | 0.08 | 0.06 | 1.44 | 0.15 | [-0.06, 0.06] |  |
|  | SES (ISEI) | 0.04 | 0.03 | 1.44 | 0.15 | [-0.07, 0.14] |  |
|  | Migration background | 0.19 | 0.05 | 3.52 | **0.00** | [-0.01, 0.10] |  |
|  | Edu: Apprenticeship | 0.37 | 0.10 | 3.75 | **0.00** | [0.08, 0.27] |  |
|  | Edu: Vocational | 0.50 | 0.10 | 4.90 | **0.00** | [0.10, 0.44] |  |
|  | Edu: Academic | 0.82 | 0.11 | 7.61 | **0.00** | [0.27, 0.63] |  |
|  | Video games | 0.09 | 0.03 | 2.85 | **0.00** | [0.55, 0.93] |  |
|  | Tobacco use | -0.05 | 0.03 | -2.13 | **0.03** | [0.08, 0.20] |  |
|  | Alcohol use | 0.03 | 0.03 | 1.18 | 0.24 | [-0.10, 0.00] |  |
|  |  |  |  |  |  |  | R^2^ = 0.206 |
|  |  |  |  |  |  |  | R^2^_adj._ = 0.196 |
| Total cognitive index | Intercept | -0.67 | 0.10 | -6.69 | **0.00** | [-0.87, -0.48] |  |
|  | Cannabis use at age 13 | -0.06 | 0.06 | -0.92 | 0.36 | [-0.17, 0.06] |  |
|  | Quadratic term | 0.00 | 0.01 | -0.01 | 0.99 | [-0.02, 0.02] |  |
|  | Cannabis use at age 11 | 0.03 | 0.04 | 0.87 | 0.38 | [-0.04, 0.11] |  |
|  | Sex | 0.12 | 0.06 | 2.11 | **0.04** | [0.01, 0.24] |  |
|  | SES (ISEI) | 0.05 | 0.03 | 1.69 | 0.09 | [-0.01, 0.11] |  |
|  | Migration background | 0.20 | 0.05 | 3.62 | **0.00** | [0.09, 0.30] |  |
|  | Edu: Apprenticeship | 0.40 | 0.10 | 4.13 | **0.00** | [0.21, 0.60] |  |
|  | Edu: Vocational | 0.50 | 0.10 | 4.86 | **0.00** | [0.30, 0.70] |  |
|  | Edu: Academic | 0.85 | 0.11 | 7.82 | **0.00** | [0.63, 1.06] |  |
|  | Video games | 0.08 | 0.03 | 2.51 | **0.01** | [0.02, 0.14] |  |
|  | Tobacco use | -0.05 | 0.03 | -1.91 | 0.06 | [-0.10, 0.00] |  |
|  | Alcohol use | 0.04 | 0.03 | 1.61 | 0.11 | [-0.01, 0.10] |  |
|  |  |  |  |  |  |  | R^2^ = 0.235 |
|  |  |  |  |  |  |  | R^2^_adj._ = 0.222 |
| Total cognitive index | Intercept | -0.58 | 0.09 | -6.42 | **0.00** | [-0.75, -0.22] |  |
|  | Cannabis use at age 15 | 0.05 | 0.04 | 1.24 | 0.22 | [-0.17, 0.08] |  |
|  | Quadratic term | -0.02 | 0.02 | -0.77 | 0.44 | [-0.04, 0.08] |  |
|  | Cannabis use at age 13 | -0.06 | 0.02 | -2.44 | **0.01** | [-0.10, 0.05] |  |
|  | Sex | 0.11 | 0.05 | 2.00 | 0.05 | [-0.04, 0.27] |  |
|  | SES (ISEI) | 0.05 | 0.03 | 1.69 | 0.09 | [-0.01, 0.14] |  |
|  | Migration background | 0.15 | 0.05 | 3.12 | **0.00** | [0.14, 0.43] |  |
|  | Edu: Apprenticeship | 0.33 | 0.09 | 3.90 | **0.00** | [-0.12, 0.37] |  |
|  | Edu: Vocational | 0.48 | 0.09 | 5.37 | **0.00** | [0.08, 0.60] |  |
|  | Edu: Academic | 0.80 | 0.10 | 8.32 | **0.00** | [0.20, 0.76] |  |
|  | Video games | 0.11 | 0.03 | 3.78 | **0.00** | [0.03, 0.19] |  |
|  | Tobacco use | -0.06 | 0.02 | -2.41 | **0.02** | [-0.10, 0.04] |  |
|  | Alcohol use | 0.04 | 0.02 | 1.81 | 0.07 | [-0.05, 0.09] |  |
|  |  |  |  |  |  |  | R^2^ = 0.230 |
|  |  |  |  |  |  |  | R^2^_adj._ = 0.220 |
| Total cognitive index | Intercept | -0.48 | 0.09 | -5.11 | **0.00** | [-0.66, -0.29] |  |
|  | Cannabis use at age 17 | -0.02 | 0.04 | -0.63 | 0.53 | [-0.10, 0.05] |  |
|  | Quadratic term | -0.03 | 0.02 | -1.20 | 0.23 | [-0.07, 0.02] |  |
|  | Cannabis use at age 15 | 0.05 | 0.03 | 1.78 | 0.08 | [0.00, 0.10] |  |
|  | Sex | 0.10 | 0.05 | 1.90 | 0.06 | [0.00, 0.21] |  |
|  | SES (ISEI) | 0.05 | 0.03 | 1.87 | 0.06 | [0.00, 0.10] |  |
|  | Migration background | 0.17 | 0.05 | 3.58 | **0.00** | [0.08, 0.27] |  |
|  | Edu: Apprenticeship | 0.22 | 0.09 | 2.47 | **0.01** | [0.05, 0.39] |  |
|  | Edu: Vocational | 0.41 | 0.09 | 4.48 | **0.00** | [0.23, 0.59] |  |
|  | Edu: Academic | 0.69 | 0.10 | 7.03 | **0.00** | [0.49, 0.88] |  |
|  | Video games | 0.10 | 0.03 | 3.61 | **0.00** | [0.05, 0.16] |  |
|  | Tobacco use | -0.05 | 0.02 | -2.02 | **0.04** | [-0.10, 0.00] |  |
|  | Alcohol use | 0.02 | 0.02 | 0.99 | 0.32 | [-0.02, 0.07] |  |
|  |  |  |  |  |  |  | R^2^ = 0.214 |
|  |  |  |  |  |  |  | R^2^_adj._ = 0.203 |
| Total cognitive index | Intercept | -0.44 | 0.10 | -4.61 | **0.00** | [-0.63, -0.25] |  |
|  | Cannabis use at age 20 | 0.01 | 0.04 | 0.23 | 0.82 | [-0.06, 0.08] |  |
|  | Quadratic term | -0.03 | 0.02 | -1.46 | 0.14 | [-0.08, 0.01] |  |
|  | Cannabis use at age 17 | -0.01 | 0.03 | -0.25 | 0.81 | [-0.06, 0.05] |  |
|  | Sex | 0.06 | 0.05 | 1.15 | 0.25 | [-0.04, 0.17] |  |
|  | SES (ISEI) | 0.05 | 0.03 | 1.70 | 0.09 | [-0.01, 0.10] |  |
|  | Migration background | 0.19 | 0.05 | 3.83 | **0.00** | [0.09, 0.28] |  |
|  | Edu: Apprenticeship | 0.19 | 0.09 | 2.12 | **0.03** | [0.01, 0.37] |  |
|  | Edu: Vocational | 0.39 | 0.10 | 4.07 | **0.00** | [0.20, 0.58] |  |
|  | Edu: Academic | 0.67 | 0.10 | 6.69 | **0.00** | [0.47, 0.87] |  |
|  | Video games | 0.13 | 0.03 | 4.27 | **0.00** | [0.07, 0.19] |  |
|  | Tobacco use | -0.05 | 0.02 | -2.21 | **0.03** | [-0.10, -0.01] |  |
|  | Alcohol use | 0.04 | 0.03 | 1.46 | 0.14 | [-0.01, 0.09] |  |
|  |  |  |  |  |  |  | R^2^ = 0.223 |
|  |  |  |  |  |  |  | R^2^_adj._ = 0.212 |
| Total cognitive index | Intercept | -0.50 | 0.09 | -5.48 | **0.00** | [-0.68, -0.32] |  |
|  | Cannabis use at age 24 | -0.01 | 0.04 | -0.34 | 0.73 | [-0.10, 0.07] |  |
|  | Quadratic term | -0.01 | 0.02 | -0.64 | 0.52 | [-0.06, 0.03] |  |
|  | Cannabis use at age 20 | 0.00 | 0.03 | -0.01 | 0.99 | [-0.06, 0.06] |  |
|  | Sex | 0.04 | 0.05 | 0.74 | 0.46 | [-0.07, 0.14] |  |
|  | SES (ISEI) | 0.05 | 0.03 | 1.75 | 0.08 | [-0.01, 0.10] |  |
|  | Migration background | 0.17 | 0.05 | 3.55 | **0.00** | [0.08, 0.27] |  |
|  | Edu: Apprenticeship | 0.27 | 0.09 | 3.10 | **0.00** | [0.10, 0.44] |  |
|  | Edu: Vocational | 0.44 | 0.09 | 4.87 | **0.00** | [0.27, 0.62] |  |
|  | Edu: Academic | 0.73 | 0.10 | 7.62 | **0.00** | [0.55, 0.92] |  |
|  | Video games | 0.14 | 0.03 | 4.79 | **0.00** | [0.08, 0.20] |  |
|  | Tobacco use | -0.05 | 0.02 | -2.09 | **0.04** | [-0.10, 0.00] |  |
|  | Alcohol use | 0.04 | 0.02 | 1.59 | 0.11 | [-0.01, 0.09] |  |
|  |  |  |  |  |  |  | R^2^ = 0.218 |
|  |  |  |  |  |  |  | R^2^_adj._ = 0.208 |

# **Table S4.** Helmert contrasts of all cognitive variables for each time point separately with covariates partialized out.

|  | Term | Estimate | *SE* | *t* | *p* | 95% CI | *η^2^_partialized_* |
| --- | --- | --- | --- | --- | --- | --- | --- |
| Sustained attention  Age 13 | Intercept | -0.06 | 0.22 | -0.29 | 0.77 | [-0.49, 0.37] |  |
|  | Category 2 vs. 1 | -0.02 | 0.07 | -0.23 | 0.82 | [-0.16, 0.13] | 0.000 |
|  | Category 3 vs. 2+1 | 0.05 | 0.09 | 0.54 | 0.59 | [-0.13, 0.23] | 0.000 |
|  | Category 4 vs. 3+2+1 | -0.17 | 0.09 | -1.94 | 0.05 | [-0.34, 0.00] | 0.004 |
|  | Category 5 vs. 4+3+2+1 | 0.07 | 0.20 | 0.33 | 0.74 | [-0.33, 0.46] | 0.000 |
| Sustained attention  Age 15 | Intercept | 0.02 | 0.06 | 0.40 | 0.69 | [-0.09, 0.14] |  |
|  | Category 2 vs. 1 | -0.04 | 0.04 | -0.94 | 0.35 | [-0.13, 0.05] | 0.001 |
|  | **Category 3 vs. 2+1** | **0.08** | **0.04** | **1.93** | **0.05** | **[0.00, 0.17]** | **0.004** |
|  | Category 4 vs. 3+2+1 | -0.04 | 0.04 | -0.84 | 0.40 | [-0.12, 0.05] | 0.001 |
|  | Category 5 vs. 4+3+2+1 | 0.02 | 0.04 | 0.52 | 0.60 | [-0.06, 0.10] | 0.000 |
| Sustained attention  Age 17 | Intercept | -0.04 | 0.04 | -0.80 | 0.43 | [-0.12, 0.05] |  |
|  | Category 2 vs. 1 | 0.01 | 0.04 | 0.34 | 0.73 | [-0.07, 0.10] | 0.000 |
|  | Category 3 vs. 2+1 | 0.00 | 0.03 | -0.01 | 0.99 | [-0.07, 0.07] | 0.000 |
|  | Category 4 vs. 3+2+1 | -0.02 | 0.03 | -0.51 | 0.61 | [-0.07, 0.04] | 0.000 |
|  | Category 5 vs. 4+3+2+1 | -0.03 | 0.03 | -1.12 | 0.26 | [-0.09, 0.02] | 0.001 |
| Sustained attention  Age 20 | Intercept | -0.05 | 0.04 | -1.20 | 0.23 | [-0.14, 0.03] |  |
|  | Category 2 vs. 1 | 0.03 | 0.04 | 0.67 | 0.50 | [-0.05, 0.11] | 0.001 |
|  | Category 3 vs. 2+1 | -0.02 | 0.04 | -0.48 | 0.63 | [-0.09, 0.05] | 0.000 |
|  | **Category 4 vs. 3+2+1** | **-0.06** | **0.03** | **-1.82** | **0.07** | **[-0.12, -0.00]** | **0.004** |
|  | Category 5 vs. 4+3+2+1 | -0.03 | 0.03 | -1.21 | 0.23 | [-0.09, 0.02] | 0.002 |
| Sustained attention  Age 24 | Intercept | 0.00 | 0.05 | -0.04 | 0.97 | [-0.09, 0.09] |  |
|  | Category 2 vs. 1 | 0.02 | 0.04 | 0.60 | 0.55 | [-0.05, 0.10] | 0.000 |
|  | Category 3 vs. 2+1 | 0.03 | 0.05 | 0.68 | 0.50 | [-0.06, 0.12] | 0.001 |
|  | Category 4 vs. 3+2+1 | -0.02 | 0.04 | -0.48 | 0.63 | [-0.09, 0.06] | 0.000 |
|  | Category 5 vs. 4+3+2+1 | -0.03 | 0.03 | -1.05 | 0.30 | [-0.08, 0.02] | 0.001 |
| Working memory  Age 13 | Intercept | -0.11 | 0.22 | -0.52 | 0.60 | [-0.54, 0.32] |  |
|  | Category 2 vs. 1 | 0.02 | 0.07 | 0.28 | 0.78 | [-0.12, 0.16] | 0.000 |
|  | Category 3 vs. 2+1 | -0.04 | 0.09 | -0.44 | 0.66 | [-0.22, 0.14] | 0.000 |
|  | Category 4 vs. 3+2+1 | -0.09 | 0.09 | -0.99 | 0.32 | [-0.26, 0.08] | 0.001 |
|  | Category 5 vs. 4+3+2+1 | -0.01 | 0.20 | -0.03 | 0.98 | [-0.40, 0.39] | 0.000 |
| Working memory  Age 15 | Intercept | 0.00 | 0.06 | -0.05 | 0.96 | [-0.12, 0.11] |  |
|  | Category 2 vs. 1 | -0.06 | 0.04 | -1.41 | 0.16 | [-0.15, 0.02] | 0.002 |
|  | Category 3 vs. 2+1 | 0.06 | 0.04 | 1.25 | 0.21 | [-0.03, 0.14] | 0.002 |
|  | Category 4 vs. 3+2+1 | -0.02 | 0.04 | -0.45 | 0.65 | [-0.10, 0.06] | 0.000 |
|  | Category 5 vs. 4+3+2+1 | 0.01 | 0.04 | 0.25 | 0.80 | [-0.07, 0.08] | 0.000 |
| Working memory  Age 17 | Intercept | 0.01 | 0.04 | 0.16 | 0.87 | [-0.08, 0.09] |  |
|  | Category 2 vs. 1 | 0.03 | 0.04 | 0.72 | 0.47 | [-0.05, 0.11] | 0.001 |
|  | Category 3 vs. 2+1 | -0.02 | 0.03 | -0.63 | 0.53 | [-0.09, 0.05] | 0.000 |
|  | Category 4 vs. 3+2+1 | 0.00 | 0.03 | 0.10 | 0.92 | [-0.06, 0.06] | 0.000 |
|  | Category 5 vs. 4+3+2+1 | 0.00 | 0.03 | 0.08 | 0.93 | [-0.06, 0.06] | 0.000 |
| Working memory  Age 20 | Intercept | 0.01 | 0.04 | 0.26 | 0.80 | [-0.07, 0.10] |  |
|  | Category 2 vs. 1 | 0.02 | 0.04 | 0.47 | 0.64 | [-0.06, 0.10] | 0.001 |
|  | Category 3 vs. 2+1 | 0.01 | 0.04 | 0.31 | 0.75 | [-0.06, 0.08] | 0.000 |
|  | Category 4 vs. 3+2+1 | 0.01 | 0.03 | 0.35 | 0.73 | [-0.05, 0.07] | 0.000 |
|  | Category 5 vs. 4+3+2+1 | 0.01 | 0.03 | 0.52 | 0.61 | [-0.04, 0.07] | 0.000 |
| Working memory  Age 24 | Intercept | 0.01 | 0.05 | 0.18 | 0.86 | [-0.08, 0.10] |  |
|  | Category 2 vs. 1 | -0.07 | 0.04 | -1.73 | 0.08 | [-0.14, 0.01] | 0.003 |
|  | Category 3 vs. 2+1 | -0.02 | 0.05 | -0.34 | 0.73 | [-0.10, 0.07] | 0.000 |
|  | Category 4 vs. 3+2+1 | 0.05 | 0.04 | 1.21 | 0.23 | [-0.03, 0.12] | 0.002 |
|  | Category 5 vs. 4+3+2+1 | 0.01 | 0.03 | 0.29 | 0.77 | [-0.04, 0.06] | 0.000 |
| Declarative memory  Age 13 | Intercept | -0.19 | 0.21 | -0.90 | 0.37 | [-0.60, 0.23] |  |
|  | **Category 2 vs. 1** | **-0.16** | **0.07** | **-2.30** | **0.02** | **[-0.30, -0.02]** | **0.006** |
|  | Category 3 vs. 2+1 | 0.02 | 0.09 | 0.24 | 0.81 | [-0.15, 0.20] | 0.000 |
|  | **Category 4 vs. 3+2+1** | **-0.23** | **0.08** | **-2.77** | **0.01** | **[-0.40, -0.07]** | **0.009** |
|  | Category 5 vs. 4+3+2+1 | 0.13 | 0.19 | 0.67 | 0.50 | [-0.25, 0.51] | 0.001 |
| Declarative memory  Age 15 | Intercept | 0.07 | 0.06 | 1.30 | 0.19 | [-0.04, 0.18] |  |
|  | Category 2 vs. 1 | 0.06 | 0.04 | 1.48 | 0.14 | [-0.02, 0.15] | 0.003 |
|  | **Category 3 vs. 2+1** | **0.09** | **0.04** | **2.10** | **0.04** | **[0.01, 0.17]** | **0.005** |
|  | Category 4 vs. 3+2+1 | 0.06 | 0.04 | 1.46 | 0.14 | [-0.02, 0.14] | 0.002 |
|  | **Category 5 vs. 4+3+2+1** | **-0.08** | **0.04** | **-2.08** | **0.04** | **[-0.15, 0.00]** | **0.005** |
| Declarative memory  Age 17 | Intercept | -0.03 | 0.04 | -0.63 | 0.53 | [-0.11, 0.06] |  |
|  | Category 2 vs. 1 | 0.02 | 0.04 | 0.51 | 0.61 | [-0.06, 0.10] | 0.001 |
|  | Category 3 vs. 2+1 | -0.04 | 0.03 | -1.19 | 0.23 | [-0.10, 0.03] | 0.002 |
|  | Category 4 vs. 3+2+1 | 0.00 | 0.03 | 0.11 | 0.91 | [-0.05, 0.06] | 0.000 |
|  | Category 5 vs. 4+3+2+1 | -0.05 | 0.03 | -1.86 | 0.06 | [-0.11, -0.00] | 0.004 |
| Declarative memory  Age 20 | Intercept | -0.01 | 0.04 | -0.22 | 0.82 | [-0.09, 0.07] |  |
|  | Category 2 vs. 1 | 0.04 | 0.04 | 1.04 | 0.30 | [-0.04, 0.12] | 0.001 |
|  | Category 3 vs. 2+1 | 0.03 | 0.04 | 0.83 | 0.41 | [-0.04, 0.10] | 0.001 |
|  | Category 4 vs. 3+2+1 | -0.01 | 0.03 | -0.32 | 0.75 | [-0.07, 0.05] | 0.000 |
|  | Category 5 vs. 4+3+2+1 | -0.04 | 0.03 | -1.35 | 0.18 | [-0.09, 0.02] | 0.002 |
| Declarative memory  Age 24 | Intercept | 0.00 | 0.05 | 0.01 | 1.00 | [-0.09, 0.09] |  |
|  | Category 2 vs. 1 | 0.00 | 0.04 | -0.13 | 0.90 | [-0.08, 0.07] | 0.000 |
|  | Category 3 vs. 2+1 | 0.03 | 0.05 | 0.70 | 0.48 | [-0.06, 0.12] | 0.001 |
|  | Category 4 vs. 3+2+1 | 0.04 | 0.04 | 0.95 | 0.34 | [-0.04, 0.11] | 0.001 |
|  | **Category 5 vs. 4+3+2+1** | **-0.07** | **0.03** | **-2.92** | **<.001** | **[-0.12, -0.02]** | **0.009** |
| Total cognitive index  Age 13 | Intercept | -0.17 | 0.22 | -0.81 | 0.42 | [-0.60, 0.25] |  |
|  | Category 2 vs. 1 | -0.08 | 0.07 | -1.06 | 0.29 | [-0.21, 0.06] | 0.001 |
|  | Category 3 vs. 2+1 | 0.01 | 0.09 | 0.16 | 0.87 | [-0.16, 0.19] | 0.000 |
|  | **Category 4 vs. 3+2+1** | **-0.23** | **0.09** | **-2.72** | **0.01** | **[-0.40, -0.06]** | **0.008** |
|  | Category 5 vs. 4+3+2+1 | 0.09 | 0.20 | 0.46 | 0.64 | [-0.30, 0.48] | 0.000 |
| Total cognitive index  Age 15 | Intercept | 0.05 | 0.06 | 0.81 | 0.42 | [-0.06, 0.16] |  |
|  | Category 2 vs. 1 | -0.02 | 0.04 | -0.36 | 0.72 | [-0.10, 0.07] | 0.000 |
|  | **Category 3 vs. 2+1** | **0.11** | **0.04** | **2.54** | **0.01** | **[0.03, 0.20]** | **0.007** |
|  | Category 4 vs. 3+2+1 | 0.00 | 0.04 | 0.11 | 0.92 | [-0.08, 0.09] | 0.000 |
|  | Category 5 vs. 4+3+2+1 | -0.02 | 0.04 | -0.66 | 0.51 | [-0.10, 0.05] | 0.000 |
| Total cognitive index  Age 17 | Intercept | -0.02 | 0.04 | -0.56 | 0.58 | [-0.11, 0.06] |  |
|  | Category 2 vs. 1 | 0.03 | 0.04 | 0.71 | 0.48 | [-0.05, 0.11] | 0.001 |
|  | Category 3 vs. 2+1 | -0.03 | 0.03 | -0.90 | 0.37 | [-0.10, 0.04] | 0.001 |
|  | Category 4 vs. 3+2+1 | 0.00 | 0.03 | -0.14 | 0.89 | [-0.06, 0.05] | 0.000 |
|  | Category 5 vs. 4+3+2+1 | -0.04 | 0.03 | -1.41 | 0.16 | [-0.10, 0.02] | 0.002 |
| Total cognitive index  Age 20 | Intercept | -0.02 | 0.04 | -0.53 | 0.60 | [-0.11, 0.06] |  |
|  | Category 2 vs. 1 | 0.04 | 0.04 | 1.09 | 0.28 | [-0.03, 0.12] | 0.001 |
|  | Category 3 vs. 2+1 | 0.01 | 0.04 | 0.33 | 0.74 | [-0.06, 0.08] | 0.000 |
|  | Category 4 vs. 3+2+1 | -0.02 | 0.03 | -0.81 | 0.42 | [-0.08, 0.03] | 0.001 |
|  | Category 5 vs. 4+3+2+1 | -0.03 | 0.03 | -0.96 | 0.34 | [-0.08, 0.03] | 0.001 |
| Total cognitive index  Age 24 | Intercept | 0.00 | 0.05 | 0.07 | 0.95 | [-0.09, 0.10] |  |
|  | Category 2 vs. 1 | -0.02 | 0.04 | -0.64 | 0.52 | [-0.10, 0.05] | 0.000 |
|  | Category 3 vs. 2+1 | 0.02 | 0.04 | 0.49 | 0.63 | [-0.07, 0.11] | 0.000 |
|  | Category 4 vs. 3+2+1 | 0.03 | 0.04 | 0.85 | 0.40 | [-0.04, 0.10] | 0.001 |
|  | **Category 5 vs. 4+3+2+1** | **-0.05** | **0.03** | **-1.80** | **0.07** | **[-0.10, 0.00]** | **0.004** |

*Note:* Category 1 = never, 2 = less than monthly, 3 = up to monthly, 4 = up to weekly, 5 = up to daily; significant results are in bold.

# **Table S5.** Pairwise comparison of the cognitive outcome variables (without and with sociodemographic and behavioral variables partialized out) using the BHC approach.

|  | Predictor | Overall test | | Early-onset, frequent users vs. Early-onset, increasing users | | Early-onset, frequent users vs. Delayed-onset, increasing users | | Early-onset, frequent users vs. Non-users and non-regular users | | Early-onset, increasing users vs. Delayed-onset, increasing users | | Early-onset, increasing users vs. Non-users and non-regular users | | Delayed-onset, increasing users vs. Non-users and non-regular users | |
| --- | --- | --- | --- | --- | --- | --- | --- | --- | --- | --- | --- | --- | --- | --- | --- |
|  |  | Chi-Square | *p* | Chi-Square | *p* | Chi-Square | *p* | Chi-Square | *p* | Chi-Square | *p* | Chi-Square | *p* | Chi-Square | *p* |
| Without sociodemographic and behavioral variables | Sustained attention | 2.38 | .50 | 1.08 | .30 | 0.79 | .37 | 2.00 | .16 | 0.12 | .73 | 0.00 | .95 | 0.48 | .49 |
|  | Working memory | 3.01 | .39 | 1.49 | .22 | 1.46 | .23 | 0.51 | .47 | 0.03 | .88 | 1.15 | .28 | 1.42 | .23 |
|  | Declarative memory | 6.36 | .10 | 1.70 | .19 | **4.23*** | **.04** | **4.99*** | **.03** | 0.94 | .33 | 1.41 | .24 | 0.02 | .90 |
|  | Total cognitive index | 4.43 | .22 | 2.65 | .10 | **4.05*** | **.04** | **4.32*** | **.04** | 0.09 | .77 | 0.05 | .83 | 0.04 | .85 |
| With sociodemographic and behavioral variables partialized out | Sustained attention | 3.00 | .39 | 0.32 | .57 | 0.01 | .93 | 0.81 | .37 | 0.47 | .49 | 0.07 | .80 | 2.29 | .13 |
|  | Working memory | 1.13 | .77 | 0.98 | .32 | 0.84 | .36 | 1.01 | .31 | 0.06 | .81 | 0.09 | .77 | 0.00 | .99 |
|  | Declarative memory | 4.79 | .19 | 0.18 | .67 | 2.19 | .14 | 2.89 | .09 | 1.32 | .25 | 1.94 | .16 | 0.03 | .87 |
|  | Total cognitive index | 4.68 | .20 | 0.87 | .35 | 1.87 | .17 | 3.75 | .05 | 0.07 | .80 | 0.58 | .45 | 0.64 | .42 |

*Note.* Estimate = * *p* <.05. Significant results are in bold.

# **Table S6.** Pairwise comparison of sociodemographic and behavioral covariates as predictors of group membership.

| Predictor | Early-onset, frequent users vs. Early-onset, increasing users | | Early-onset, frequent users vs. Delayed-onset, increasing users | | Early-onset, frequent users vs. Non-users and non-regular users | | Early-onset, increasing users vs. Delayed-onset, increasing users | | Early-onset, increasing users vs. Non-users and non-regular users | | Delayed-onset, increasing users vs. Non-users and non-regular users | |
| --- | --- | --- | --- | --- | --- | --- | --- | --- | --- | --- | --- | --- |
|  | Estimate (*SE*) | *OR* | Estimate (*SE*) | *OR* | Estimate (*SE*) | *OR* | Estimate (*SE*) | *OR* | Estimate (*SE*) | *OR* | Estimate (*SE*) | *OR* |
| Sex^1^ | -0.87 (0.86) | 0.42 | **-1.91* (0.79)** | 0.15 | **-2.41** (0.74)** | 0.09 | **-1.04* (0.50)** | 0.35 | **-1.54*** (0.43)** | 0.22 | -0.50 (0.30) | 0.61 |
| Migration Background^2^ | **-1.51* (0.69)** | 0.22 | -1.23 (0.65) | 0.29 | **-1.62** (0.62)** | 0.20 | 0.28 (0.40) | 1.32 | -0.12 (0.35) | 0.89 | -0.39 (0.26) | 0.68 |
| ISEI^3^ | 0.70 (0.40) | 2.02 | **0.76* (0.37)** | 2.13 | 0.22 (0.35) | 1.25 | 0.05 (0.22) | 1.06 | **-0.48* (0.20)** | 0.62 | **-0.53*** (0.15)** | 0.59 |
| Edu: Apprentice^4^ | -0.33 (0.78) | 0.72 | -0.08 (0.73) | 0.93 | 0.53 (0.70) | 1.70 | 0.26 (0.57) | 1.29 | 0.86 (0.49) | 2.37 | 0.61 (0.46) | 1.84 |
| Edu: Vocational^4^ | -0.64 (0.87) | 0.53 | -0.38 (0.81) | 0.69 | 0.41 (0.76) | 1.51 | 0.26 (0.64) | 1.30 | 1.05 (0.55) | 2.86 | 0.79 (0.49) | 2.20 |
| Edu: Academic^4^ | 0.02 (1.16) | 1.02 | 0.75 (1.10) | 2.11 | 1.42 (1.07) | 4.13 | 0.73 (0.69) | 2.08 | **1.40* (0.61)** | 4.06 | 0.67 (0.52) | 1.96 |
| Video Games^5^ | 0.11 (0.26) | 1.12 | 0.31 (0.24) | 1.36 | -0.13 (0.23) | 0.88 | 0.20 (0.19) | 1.22 | -0.24 (0.17) | 0.79 | **-0.44*** (0.13)** | 0.64 |
| Tobacco^5^ | 0.06 (0.32) | 1.06 | 0.52 (0.31) | 1.69 | -0.52 (0.27) | 0.60 | **0.47* (0.21)** | 1.59 | **-0.58*** (0.17)** | 0.56 | **-1.04*** (0.16)** | 0.35 |
| Alcohol^5^ | 0.24 (0.27) | 1.28 | 0.21 (0.25) | 1.24 | 0.00 (0.22) | 1.00 | 0.03 (0.20) | 1.03 | -0.24 (0.18) | 0.78 | -0.21 (0.13) | 0.81 |
| *Note.* Estimate = *β* from R3STEP analysis; *** *p* <.001; ** *p* <.01; * *p* <.05. ^1^: 0 = female, 1 = male; ^2^: 0 = migration background, 1 = at least one parent born in Switzerland; ^3^: ISEI range 16-90 z-standardized; ^4^: reference category = Below apprenticeship, ^5^: 1-7 range z-standardized. | | | | | | | | | | | | |

# **Figure S1.** Missingness analysis.

*Note*: Correlation heatmap of missing cognitive data (1 = missing, 0 = observed) with cannabis-use variables (ages 11, 13, 15, 17, 20, and 24). Correlations were estimated using two-sided Pearson tests; significance is indicated as ***p < .001, *p < .01, p < .05. As shown in the lower left part of the matrix, missingness on the four cognitive outcomes was essentially unrelated to cannabis use at any age (r from −.01 to .05), indicating no strong or systematic selective attrition with respect to cannabis. By contrast, the cannabis-use measures were moderately to strongly intercorrelated across waves (r ≈ .22–.68), reflecting stability and escalation of use over time.

# **Figure S2.** Alluvial diagram of cannabis use.

# **Figure S3.** Correlations of all variables.

*Note:* Only significant correlations are displayed in the matrix; non-significant values are left blank, * = <.05, ** = <.01, *** = <.001.

# **Figure S4.** Correlations of all variables with covariates partialized out.

*Note:* Only significant correlations are displayed in the matrix; non-significant values are left blank, * = <.05, ** = <.01, *** = <.001.

# **Figure S5.** Boxplots of cannabis use between ages 11 and 24 and the cognitive variables.

# **Figure S6.** Quadratic regressions with sociodemographic and behavioral variables as covariates.

*Note:* “_Q” stands for the quadratic term. The quadratic model at age 11 did not converge due to a small number of cannabis users. Dots represent standardized regression coefficients (β), with 95% confidence intervals (CIs) shown as horizontal lines. Blue indicates statistically significant predictors (*p* < .05); grey indicates non-significant effects.
